# *proRate*: an R package to infer gene transcription rates with a novel least sum of squares method

**DOI:** 10.1093/nargab/lqaf123

**Published:** 2025-09-05

**Authors:** Yu Liu, Fadhl Alakwaa

**Affiliations:** Department of Computational Medicine and Bioinformatics, University of Michigan, Ann Arbor 48109 MI, United States; Department of Internal Medicine, Nephrology Division, University of Michigan, Ann Arbor 48109 MI, United States

## Abstract

The dynamics of transcriptional elongation influence many biological activities, such as RNA splicing, polyadenylation, and nuclear export. To quantify the elongation rate, a typical method is to treat cells with drugs that inhibit RNA polymerase II (Pol II) from entering the gene body and then track Pol II using Pro-seq or Gro-seq. However, the downstream data analysis is challenged by the problem of identifying the transition point between the gene regions inhibited by the drug and not, which is necessary to calculate the transcription rate. Although the traditional hidden Markov model (HMM) can be used to solve it, this method is complicated with its hidden variable and many parameters to be estimated. Hence, we developed the R package *proRate*, which identifies the transition point with a novel least sum of squares (LSS) method and calculates the elongation rate accordingly. In addition, *proRate* also covers other functions frequently used in transcription dynamic study, including metagene plotting, pause index calculation, gene structure analysis, etc. The effectiveness of this package is proved by its performance on three Pro-seq or Gro-seq datasets, showing higher accuracy than HMM. *proRate* is freely available at https://github.com/yuabrahamliu/proRate or https://github.com/FADHLyemen/proRate.

## Introduction

The dynamics of transcriptional elongation influence various post-transcriptional processes, such as splicing, polyadenylation, and nuclear export [1–5]. For example, in Drosophila and mammalian cells, a slow RNA polymerase II (Pol II) mutant results in defects in Pol II processivity and messenger RNA (mRNA) polyA site selection [6, 7].

To quantify the elongation rate, a typical method is to treat cells with drugs able to inhibit RNA Pol II from entering the gene body, such as 5,6-dichloro-1-β-d-ribofuranosylbenzimidazole (DRB), and then track Pol II using Pro-seq or Gro-seq [8, 9]. As the DRB-dependent blocking of Pol II entry persists, a read blank region will form on the gene body because few Pol II enter it. On the other hand, downstream of this blank region (Pol II depleted state) is the intact read region (Pol II occupied state) formed by the unaffected Pol II having entered the gene before the drug blocking. Hence, the length of the blank region is considered the distance Pol II should have gone through during the blocking period, and the corresponding Pol II transcription rate can be calculated based on this distance and the blocking time.

To get the distance, the transition point between the Pol II depleted state and the intact state must be identified. Traditionally, a 2-state hidden Markov model (HMM) is used to solve this problem [8]. However, this method is complicated with many parameters to be estimated and the model’s hidden variable to be solved by the expectation–maximization (EM) iteration [10]. In addition, it typically assumes that the observed continuous data follow a normal distribution, which cannot always be fulfilled. Hence, we tried to solve the problem using a different method and developed the R package *proRate*. It identifies the transition point via a novel least sum of squares (LSS) method, which is more efficient than HMM and has no requirement on the data distribution. In addition, *proRate* also provides other functions, including metagene analysis, pause index calculation, gene structure analysis, etc. Testing this package on three datasets shows the accurate performance of our LSS method. Compared with the traditional HMM model, it identifies the Pol II-depleted-occupied states transition points from more genes and achieves higher accuracy. We believe *proRate* can become a helpful tool for transcription dynamic research.

## Materials and methods

### Package overview

**Figure 1.**
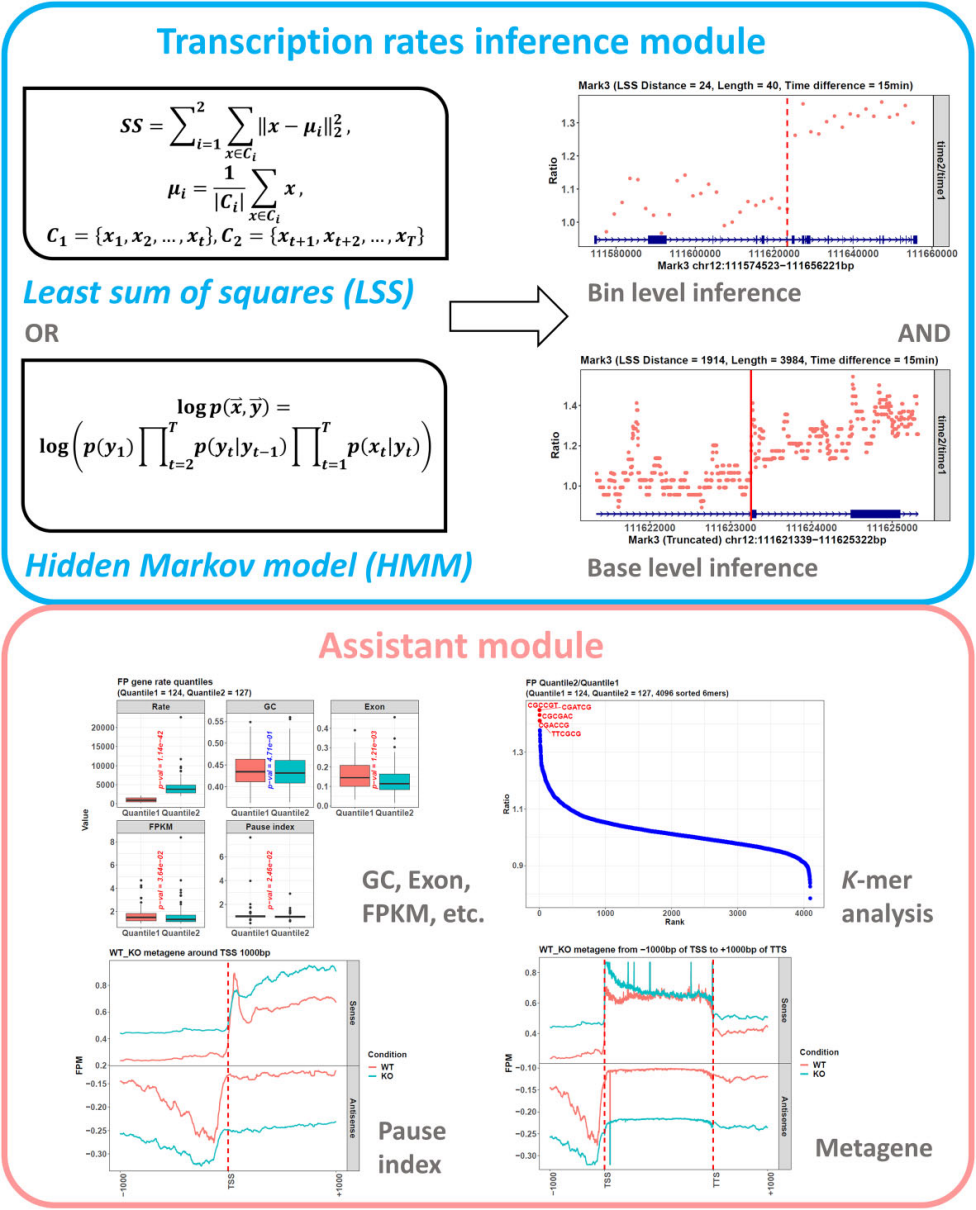
Package modules. The *proRate* package contains two modules: the rates inference module and the assistant module.

The package contains two modules (Fig. 1). The first is the transcription rates inference module. Its functions, *calrate* and *mcalrate*, accept the Pro-seq or Gro-seq data from transcription inhibition experiments, which depend on Pol II inhibitors such as DRB. Then, they can construct an LSS model or a traditional HMM model to identify the genes’ Pol II-depleted-occupied transition points and calculate the genes’ transcription rates.

The second module is the assistant module. It provides functions frequently used in transcription dynamic studies. For example, the functions *metaplot*, *mmetaplot*, and *plotprocessing* perform metagene analysis; the function *calpauseidx* calculates a gene’s pause index, which is the ratio of the Pol II density between its promoter and its gene body and is closely related to its transcription rate [11]; and the functions *getgc*, *getkmer*, and *getexon* can analyze gene structure and reveal its influence on transcription dynamics.

### Transcription rates inference

The function *calrate* in our package was used to infer gene transcription rates. It read in a pair of Pro-seq or Gro-seq bam files: the drug treatment and reference files. The former was from inhibiting transcription for a specific time, and the latter was untreated. Then, *calrate* divided each gene into 40 bins. For each bin, the normalized read count ratio between the treatment and the reference files was calculated, so a vector with 40 ratios was generated for a gene. After that, a pointer was inserted into it, dividing the 40 bins into two vector segments. Then, the variance of each segment’s bin ratios was calculated and summed up as the whole vector’s sum of squares (SS) value. Because the pointer could be inserted through the first bin to the 40th, a total of 40 SS values could be obtained, and the smallest one corresponded to the treatment file’s depleted/intact transition point. Next, the Wilcoxon test was used to check this point’s statistical significance. At this stage, the point was a bin, including hundreds of bases. Therefore, this bin and its downstream neighbor were further merged and expanded to the single-base level, and the LSS method was used on this region with a pointer going through it base by base. This time, the smallest SS corresponded to the single-base transition point. More details of our LSS method were described in Supplementary Data. In addition, *calrate* could perform the HMM method, which also inferred the rates in the bin-expansion two-stage manner, but the transition point for each stage was calculated via HMM.

It should be demonstrated that *calrate*’s LSS and HMM methods were conducted in the bin-expansion framework, i.e. before inferring on the base level, they needed to be calculated on the bin level first. There were two reasons to adopt this two-stage framework, rather than performing LSS on the whole genes’ bases directly. One was to accelerate the computational speed, and the other was to improve the accuracy.

In the case that LSS was used directly on a gene with *n* base pairs, the pointer needed to be inserted through all its *n* base pairs, so there would be *n* corresponding SS values to be calculated for the minimum SS and the transition point identification. However, in the bin-expansion framework, the gene was first divided into *m* bins, with each bin containing *n*/*m* base pairs. As the pointer first went through the *m* bins, and then the following two expanded merged bins with 2 × (*n*/*m*) base pairs, a total of *m* + 2 × (*n*/*m*) corresponding SS values would be calculated. In most cases, rate inference would be performed on genes with sufficient length, such as a gene with 40 000 bp (*n* = 40 000), and the bin number was usually chosen as 40 (*m* = 40), which was the default value in the *calrate* function. Hence, by conducting LSS directly on the *n* base pairs, a total of *n* = 40 000 SS values would be calculated. In contrast, with the bin-expansion framework, only *m* + 2 ×(*n*/*m*) = 40 + 2 ×(40 000/40) = 2040 SS values needed to be calculated. This largely reduced the computational burden and improved the speed.

In addition, when the gene length *n* was as long as 40 000 bp, going through all of them would meet more noises, and the pointer would be trapped in a local minimum more easily. However, the bin-division step summarized many base pairs into one bin, which could offset many noises, and relieve the local minimum dilemma. In this case, the binning step improved the inference accuracy. Hence, given the positive effects on the computational speed and accuracy, the function *calrate* introduced the bin-expansion framework into the rate inference.

## Results

### LSS infers the Paf1C-regulated transcription rates more accurately than HMM

To test the performance of *proRate*, we first applied it to the Gene Expression Omnibus (GEO) dataset GSE116169 [8]. It studied the transcriptional elongation factor Paf1C and conducted DRB-dependent transcription inhibition on two kinds of mouse C2C12 myoblasts: the wildtype (WT) cells with normal Paf1C expression and the ones with Paf1C conditionally knocked out (KO). The WT and KO cells were treated with DRB for 15 min (the WT15 and KO15 cell groups) or left untreated (the WT0 and KO0 cell groups). Then, paired-end Pro-seq was used to sequence their nascent RNAs, and the directional ligation method was used to distinguish sense and anti-sense gene strands. Because of DRB’s exclusion effect on Pol II, the WT15 and KO15 cells would have a read-depleted region on their gene bodies, while that of WT0 and KO0 cells were intact.

**Figure 2.**
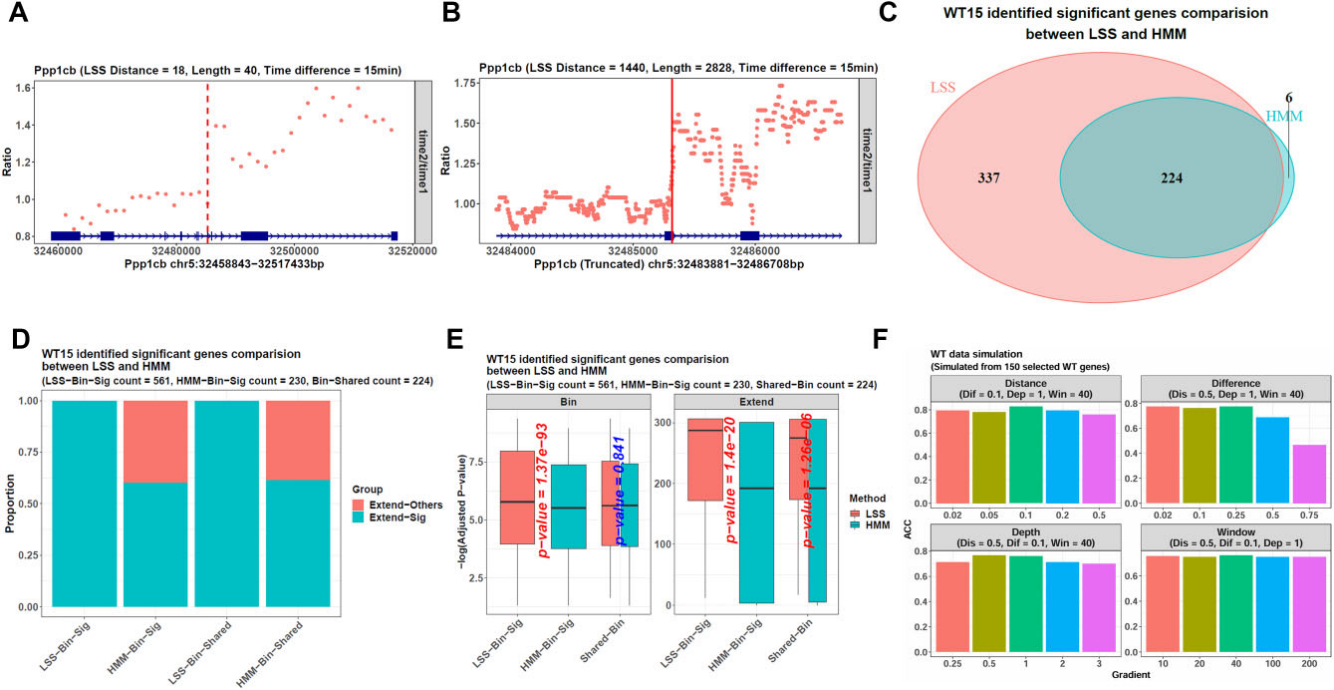
LSS infers the mouse C2C12 cell transcription rates more accurately than HMM. (**A**) In the WT15 data, *calrate* identifies the gene Ppp1cb’s depleted/intact transition point in a bin-expansion framework. On the bin level, it identifies the 18th bin with the LSS method. The dots represent the normalized read count ratios between the WT15 and WT0 bins. The vertical dotted line labels the identified 18th bin. (**B**) On the base level, *calrate* expands the 18th and 19th bins and uses LSS in this region. The dots represent the normalized read count ratios between the WT15 and WT0 bases. It identifies the base 1440 as the final point, as indicated by the vertical solid line. This point is the base 26 478 of the whole gene Ppp1cb, whose length is 58 591 bp. (**C**) In the WT15 data, LSS finds a significant transition point in 561 genes with a length ≥40 kb and an FPKM >1 in the WT0 reference. On the other hand, HMM identifies a significant point in 230 genes. Among them, 224 genes are shared. (**D**) All the LSS and HMM transition points are significant on the bin level. However, if checking them in the expanded regions on the base level, all the LSS points in the 561 genes (the LSS-Bin-Sig point group) are still significant. In comparison, the HMM points in the 230 genes (the HMM-Bin-Sig point group) only contain 139 significant ones with a significance proportion of 139/230 = 0.604. Correspondingly, for the 224 shared genes, their points identified by LSS (the LSS-Bin-Shared point group) are all significant on the base level. However, the ones identified by HMM (the HMM-Bin-Shared point group) only contain 138 significant ones with a significance proportion of 138/224 = 0.616. The cyan parts represent the point groups’ significance proportions, and the red ones represent insignificance proportions. (**E**) Although all the identified points are significant on the bin level, the 561 LSS points (LSS-Bin-Sig points) have much smaller significance *P*-values than the 230 HMM ones (HMM-Bin-Sig points), i.e. they are much larger on the minus logarithm scale (the facet Bin). If checking the base level (the facet Extend), the LSS-Bin-Sig points also have smaller significance *P*-values, and in the 224 shared genes (Shared-Bin genes), their transition points identified by LSS also have much smaller *P*-values. (**F**) The simulated datasets are generated from the WT0 data and have pre-defined gene transition points. Their inference results show that LSS’s accuracy is influenced by the depleted region’s length (the parameter Distance), the intact region’s depth (the parameter Depth), the FPKM ratio between these regions (the parameter Difference), and the bin number used in the bin-expansion framework (the parameter Window). When the Distance value is fixed as 0.5, the Depth value is fixed as 1, and the Window value is fixed as 40 (the top-right facet), i.e. the depleted region’s length is 50% of the corresponding gene, the intact region’s sequencing depth is the same as the original WT0 data, and the gene is divided into 40 bins in the bin-expansion framework, LSS’s accuracy decreases sharply from 0.773 to 0.467 as the Difference parameter increases from 0.02 to 0.75, i.e. as the depleted region’s FPKM increases from 2% to 75% of the intact one. On the other hand, when fixing the Difference value as 0.1, the Depth value as 1, and the Window value as 40 (the top-left facet), the Distance parameter influences LSS’s accuracy independently, as its increase from 0.02 to 0.1 leads to the accuracy’s increase from 0.793 to 0.827. In addition, the parameters Depth and Window also show their effects because, in the condition of Distance = 0.5, Difference = 0.1, and Window = 40 (the bottom-left facet), LSS’s accuracy increases from 0.713 to 0.76 as the parameter Depth increases from 25% of the original WT0 (Depth = 0.25) to the same as WT0 (Depth = 1). In the condition of Distance = 0.5, Difference = 0.1, and Depth = 1 (the bottom-right facet), LSS’s accuracy increases from 0.753 to 0.76 as the parameter Window increases from 10 to 40.

We used the LSS method on the WT15 group to infer its gene transcription rates, which was fulfilled by the function *calrate* in our package. We used *calrate*’s bin-based strategy and the inferred genes were the ones with a length ≥40 kb and an FPKM (fragments per kilobase million) >1 in the matched WT0 reference. LSS identified 561 genes with a significant transition point, such as Ppp1cb, whose length was 58 591 bp. In the bin-expansion framework, LSS first identified its 18th bin as the transition point (Fig. 2A). Then, it expanded this bin and its downstream neighbor and identified the base 1440 in this region (Fig. 2B). Hence, the final transition point was the base 26 478 of the whole gene.

On the other hand, HMM identified a significant transition point in 230 genes of the WT15 group, less than LSS’s 561. Among them, 224 genes were shared by the two methods (Fig. 2C). In most cases, the significant points from LSS and HMM differed, even if they were in the same gene. Moreover, it was noteworthy that the Wilcoxon test judged the points’ significance when they were still bins in the bin-expansion framework. Therefore, all the identified transition points were significant on the bin level. However, if checking them in the expanded regions on the base level, a difference could be caught: all the 561 LSS points were still significant, but the 230 HMM points only contained 139 significant ones. Hence, the base-level significance proportion was 1 for LSS but only 139/230 = 0.604 for HMM (Fig. 2D). In addition, if comparing the points’ significance *P*-values directly, it could be observed on the base level that the LSS *P*-values were significantly smaller than HMM, i.e. their minus logarithm values were larger (Fig. 2E). Furthermore, even on the bin level, LSS *P*-values were also smaller. Hence, although the former conclusion indicated the significance of all the points on the bin level, the LSS ones’ smaller *P*-values showed that they were more accurate.

These advantages of LSS could be demonstrated with an example gene, Cemip2, in the WT15 group. LSS and HMM identified different transition points in Cemip2, and both were significant at both the bin and base levels (Supplementary Fig. S1). However, further comparison showed that the LSS point’s *P*-values were much smaller than those of HMM (LSS’s adjusted *P*-value on bin = 5.18e-7 < HMM’s 2.78e-4, LSS’s adjusted *P*-value on base = 0 < HMM’s 3.66e-280). Hence, the LSS transition point was more significant than HMM. The reason that they identified different points was their methods’ differences. LSS found the point that minimizes the SS, or mean squared error (MSE), of the gene. On the other hand, HMM found the point that would be correct for the maximum-likelihood model of the gene’s Markov process. In this case, HMM’s point had the greatest probability of generating the gene’s read count ratios observed. However, such an HMM point with the highest probability was not necessarily the LSS point with the smallest MSE. In addition, HMM’s probability calculation depended on the assumption that the read count ratios followed a normal distribution, which could not always be fulfilled and then brought errors to the probability estimation. Hence, the two methods identified different transition points in the same gene, and LSS’s point was more significant.

Furthermore, we also compared LSS’s results with another two computational tools. The first one was *groHMM*, which was also an HMM-based algorithm, and it decoded the genome into transcribed and nontranscribed states [12]. When used on a DRB-blocked gene in the WT15 data, the transition point between these two states also indicated the gene transcription distance. The second tool was *MACS2*, which was originally a ChIP-seq peak caller to identify read peaks across the genome from two files: the reference and the ChIP-seq treatment one [13]. However, since the transcription rate inference here was also based on a reference and a treatment file, we transferred *MACS2* to this task and the point it identified between the read peak and the neighbor region was also the transcription rate transition point.

It was noteworthy that *groHMM* and *MACS2*’s calculation did not include the bin-expansion framework as LSS. Hence, to compare their results on both the bin and base levels, we manually converted their returned points into bin and expansion-base coordinates, using LSS’s framework. The final results showed that *groHMM* and *MACS2* identified 152 and 85 genes with a significant bin-level point, respectively. Both of them were less than LSS’s 561 (Supplementary Fig. S2A and B). Among them, *groHMM* had 148 genes shared with LSS, and *MACS2* had 82, i.e. both *groHMM* and LSS identified a significant bin-level point in the 148 genes, and both *MACS2* and LSS identified such a point in the 82 genes.

Further comparison on base-level significance still confirmed the higher accuracy of LSS. Although 150 of the 152 *groHMM* points were base-level significant (significance proportion was 150/152 = 0.987), and 72 of the 85 *MACS2* points were also base-level significant (significance proportion was 72/85 = 0.847), their performance was still weaker than LSS with all the 561 points as base-level significant (Supplementary Fig. S2C and D). Meanwhile, the LSS points had smaller significance *P*-values on the base level than *groHMM* and *MACS2* (Supplementary Fig. S2E and F). Hence, LSS performed not only better than the HMM method of our *calrate* function but also better than the public tools *groHMM* and *MACS2*.

In addition to the WT15 sample above, we also tested the LSS, HMM, *groHMM*, and *MACS2*’s performance on the KO15 group, with KO0 as its reference. The result also showed that LSS identified a significant transition point in more genes, and its points were more significant than other methods on both the bin and base levels (Supplementary Figs S3 and S4).

Then, we further quantified the accuracy of LSS. As described in Supplementary Data, we selected 150 genes ≥40 kb with an FPKM >1 from the WT0 data. Then, we constructed several simulated Pro-seq datasets from them, and all their genes had a pre-defined transition point. During the simulation, we changed three parameters to generate various dataset conditions. They could have different sequencing depths (the parameter Depth) and depleted region lengths (the parameter Distance). In addition, the FPKM ratios between their genes’ depleted regions and intact regions could also be different (the parameter Difference). After conducting LSS, if the identified transition point were ≤50 bp from the true point, we would consider it correct. Hence, for a simulated dataset, the LSS accuracy out of all its genes could be calculated (Fig. 2F). The result showed that all three parameters independently influenced the accuracy, especially the depleted/intact FPKM ratio. LSS had the highest accuracy of 0.773 when the ratio was 0.02, i.e. the depleted region’s FPKM value was only 2% of the intact one. Then, as this ratio increased, the accuracy decreased. When the ratio reached 0.75, which meant the depleted region’s FPKM was close to the intact one, the accuracy reached its minimum of 0.467. This case corresponded to the experimental condition that when the DRB used to inhibit the transcription was insufficient, most Pol II would still enter the gene body, and the depleted region would have a similar FPKM value to the intact. Finally, the transition point could not be identified accurately. In addition to the FPKM ratio, the depleted region length also affected LSS’s accuracy. When a dataset had a value of 0.02, i.e. all of its genes’ depleted region lengths were 2% of the whole gene lengths, the LSS accuracy was 0.793. Then, as the length gradually increased to 0.1, the accuracy increased to 0.827. However, a further increase until 0.5 would lead to an accuracy decrease to 0.76. On the other hand, when a dataset’s intact regions kept all their reads in the original WT0 file, i.e. the sequencing depth was 1, the LSS accuracy was 0.76 in the dataset. However, if these regions only kept 25% of their original WT0 reads, i.e. the depth value became 0.25, the accuracy would be weakened to 0.713. Hence, LSS’s accuracy could be influenced by the depleted region’s length, the intact region’s depth, and the FPKM ratio between these regions.

After checking these conditions of the dataset itself, we tested the parameter *window_num* of our function *calrate*. We found that when the dataset conditions were fixed, i.e. the three parameters above were fixed (Distance = 0.5, Difference = 0.1, Depth = 1), the algorithm parameter *window_num* could also influence LSS’s accuracy via the bin-expansion framework. It defined the number of bins to be divided for a gene, and its default value was 40. However, we set it to different numbers so that a gene would be divided into 10, 20, 40, 100, and 200 bins in the framework, and LSS’s accuracy changed with this number. Its highest value was 0.76 when *window_num* was set to 40, the default value. At the same time, other bin numbers made the accuracy decrease, but the lowest value was still 0.747 when *window_num* was 20, 100, and 200. Hence, the default value of 40 had a slight advantage.

In addition to LSS, we also tried to explore the effects of these parameters on HMM’s accuracy. However, most transition points identified by it were >50 bp from the true one, leading to very low accuracy. Hence, we did not further study the HMM case.

Furthermore, using the selected 150 genes from the WT0 data, we also constructed a simulated negative dataset, i.e. all of its genes had no read-depleted region or transition point, which corresponded to the experimental condition that DRB was not used in the treatment group. Hence, LSS should detect no significant transition point. However, if it detected any, these points would be the negative ones reported as positive by mistake, i.e. they were the false positive ones. In this case, LSS would have a false positive rate >0, and a true negative rate <1. However, the result on this negative dataset showed that, as the *window_num* parameter changed from 10 to 200, LSS consistently detected no false positive points, so it achieved a false positive rate of 0 and a true negative rate of 1, reflecting its excellent performance in excluding false positive results (Supplementary Fig. S5).

**Figure 3.**
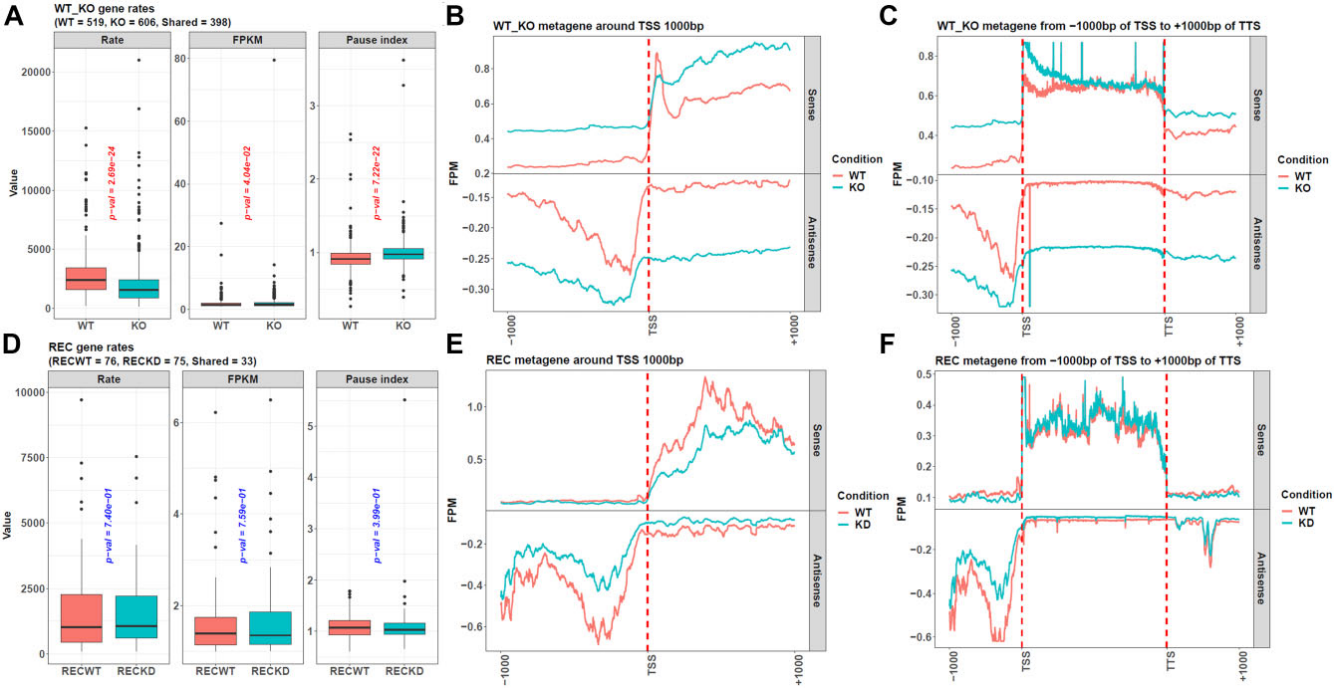
The package detects the influences of Paf1C and RecQL5 on gene transcription. (**A**) From the results of *calrate*, the Paf1C KO cells have significantly smaller transcription rates than the WT ones, but their genes’ FPKMs are much larger. Meanwhile, the function *calpauseidx* shows that the KO genes’ pause indices are also significantly larger, hinting that their Pol II tends to pause at the promoter regions rather than entering the gene bodies to perform transcription. (**B**, **C**) The functions *mmetaplot* and *plotprocessing* generate the WT and KO cells’ metagenes. It shows that the KO one has much larger nascent RNA FPM values around its transcription start site (TSS) (**B**), but in the downstream gene body region, it is similar to WT (**C**). (**D**) From *calrate*, the RECKD cells with RecQL5 knocked down have faster transcription rates than the RECWT ones, but their genes’ FPKMs are smaller. In addition, *calpauseidx* shows that the RECKD genes’ pause indices are smaller, indicating that their Pol II tends to enter the gene bodies to conduct transcription. (**E**, **F**) The functions *mmetaplot* and *plotprocessing* show that the RECKD’s metagene has smaller nascent RNA FPM values around its TSS (**E**), but in the downstream gene body region, it is similar to RECWT (**F**). All the results are based on genes with a length ≥40 kb and an FPKM >1 in the reference cells. Furthermore, because a gene’s pause index is calculated as the ratio of the Pol II density 1 kb around its TSS to its gene body, it will be excluded if this gene overlaps with others in these regions. Finally, 519 genes in the WT cells, 606 genes in the KO cells, 76 genes in the RECWT cells, and 75 genes in the RECKD cells are analyzed. In addition, *mmetaplot* compresses all the gene regions from TSS to transcription termination site to 2 kb to unify, so they can be aligned when plotting the metagene. To overcome the outlier gene sites with an extremely large FPM, *plotprocessing* defines the 99% quantile of the metagene FPMs in panels (B), (C), (E), and (F) as their maximum values and any sites with a larger value will be reduced.

From the results above, in most simulated conditions, LSS achieved an accuracy of around 0.8, so we considered this method reliable and explored its results on the WT15 and KO15 cells. After inferring the transition points with LSS, *proRate* calculated the transcription rates from the depleted region lengths and the DRB treatment time, and its returned results also contained other information, such as the genes’ FPKMs in the references WT0 and KO0. In addition, we used another function in our package, *calpauseidx*, to get the genes’ pause indices in WT0 and KO0. A comparison showed that the KO cell genes had a median transcription rate of 1565.5 bp/min, much slower than the WT one of 2420.667 bp/min (Fig. 3A). Hence, the Paf1C factor knockout reduced their genes’ transcription rates, consistent with the original study’s conclusion [8]. Correspondingly, the KO cell genes also showed a significantly larger pause index, indicating most Pol II paused at the promoter regions rather than entering the gene bodies to perform transcription, which explains the KO cells’ slow transcription. In addition, the KO cell genes had a much larger FPKM value. This was supported by the results from the functions, *mmetaplot* and *plotprocessing*, in our package. Their metagene plots showed that the Paf1C knockout increased the nascent RNAs around the promoter, but those in the downstream gene body were similar to the WT ones, so the KO genes had an increased overall FPKM, and their pause indices also became larger (Fig. 3B and C).

This case study showed the accurate performance of LSS in the Paf1C-relevant data.

### LSS infers the RecQL5-regulated transcription rates more accurately than HMM

Next, we applied the package to another GEO dataset, GSE49133 [14], which included two kinds of human HEK293T-Rex cells, i.e. the ones with the elongation factor RecQL5 knocked down (RECKD cells) and the ones with normal RecQL5 expression (RECWT cells). DRB was used on them to inhibit transcription for 25 min. Then, single-end Gro-seq data were prepared for the knockdown and WT cells (RECKD25 and RECWT25 Gro-seq data). In addition, the cells without DRB treatment were also sequenced to generate references (RECKD0 and RECWT0 data). After preprocessing, their bam files only kept the reads with a mapping quality score ≥10.

**Figure 4.**
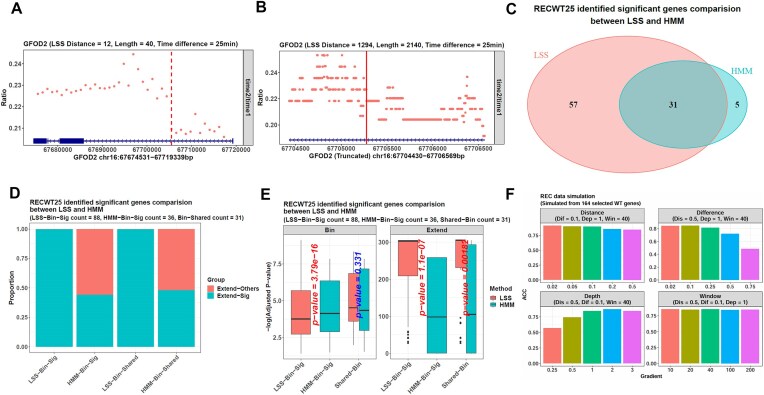
LSS infers the human HEK293T-Rex cell transcription rates more accurately than HMM. (**A**) In the RECWT25 data, *calrate* identifies the gene GFOD2’s transition point in a bin-expansion manner. It identifies the 12th bin with the LSS method on the bin level. The dots represent the normalized read count ratios between the RECWT25 and RECWT0 bins. The vertical dotted line indicates the transition bin. (**B**) On the base level, *calrate* expands the 12th and 13th bins and uses LSS in this region. The dots represent the normalized read count ratios between the RECWT25 and RECWT0 bases. It identifies the base 1294 as the final point, as labeled by the vertical solid line. This point is also the base 14 064 of the whole gene GFOD2, which is 44 809-bp long. (**C**) In the RECWT25 data, LSS finds a significant transition point in 88 genes with a length ≥40 kb and an FPKM >1 in the reference. On the other hand, HMM identifies a significant point in 36 genes. Among them, 31 are shared by the two methods. (**D**) All the LSS and HMM points are significant on the bin level. However, on the base level, all the LSS points in the 88 genes (the LSS-Bin-Sig point group) are still significant, but the HMM points in the 36 genes (the HMM-Bin-Sig point group) only contain 16 significant ones with a significance proportion of 16/36 = 0.444. Furthermore, for the 31 shared genes, their points identified by LSS (the LSS-Bin-Shared point group) are all significant on the base level. However, those identified by HMM (the HMM-Bin-Shared point group) only contain 15 significant ones with a significance proportion of 15/31 = 0.484. The cyan parts represent the point groups’ significance proportions, and the red ones represent insignificance proportions. (**E**) Although all the identified points are significant on the bin level, the 36 HMM-Bin-Sig points have much smaller significance *P*-values than the 88 LSS-Bin-Sig points (the facet Bin). However, if checking the base level (the facet Extend), the LSS-Bin-Sig points have smaller significance *P*-values than the HMM-Bin-Sig ones, and for the 31 shared genes (Shared-Bin genes), their transition points identified by LSS also have much smaller *P*-values. (**F**) The simulated datasets are generated from the RECWT0 data and have pre-defined gene transition points. Their inference results show that LSS’s accuracy is influenced by the depleted region’s length (the parameter Distance), the intact region’s depth (the parameter Depth), the FPKM ratio between these regions (the parameter Difference), and the bin number used in the bin-expansion framework (the parameter Window). When the Distance value is fixed as 0.5, the Depth value is fixed as 1, and the Window value is fixed as 40 (the top-right facet), i.e. the depleted region’s length is 50% of the corresponding gene, the intact region’s sequencing depth is the same as the original RECWT0 data, and the gene is divided into 40 bins in the bin-expansion framework, LSS’s accuracy decreases sharply from 0.843 to 0.484 as the Difference parameter increases from 0.02 to 0.75, i.e. as the depleted region’s FPKM increases from 2% to 75% of the intact one. On the other hand, the parameter Depth also shows an effect because, in the condition of Distance = 0.5, Difference = 0.1, and Window = 40 (the bottom-left facet), LSS’s accuracy increases from 0.568 to 0.872 as the intact region’s depth increases from 25% to 200% of the original RECWT0 (from Depth = 0.25 to Depth = 2). In addition, the parameters Distance and Window can also influence LSS’s accuracy independently when fixing other parameters (the top-left and bottom-right facets).

Then, we used our package on genes with a length ≥40 kb and an FPKM >1 in the reference files, and the results on RECWT25 showed that this task was difficult because LSS only identified a significant point in 88 genes, such as GFOD2. On the bin level, LSS identified its 12th bin as the transition point (Fig. 4A), and on the base level, it identified the base 1294 of the expanded region, which was also the base 14 064 of the whole gene with a length of 44 809 bp (Fig. 4B).

Although LSS only found 88 genes’ transition points, it was still better than HMM, which only identified a point in 36 genes, and among them, 31 were shared by the two methods (Fig. 4C). All the points were significant on the bin level and all the LSS ones were also significant on the base level. In contrast, only 16 of the 36 HMM points were base-level significant, i.e. their significance proportion was 16/36 = 0.444 (Fig. 4D). Furthermore, a direct comparison of the significance *P*-values showed that LSS points had much smaller base-level *P*-values (Fig. 4E). On the other hand, the 36 HMM points showed smaller bin-level *P*-values than the 88 LSS ones. However, given the larger significant points number and the smaller base-level *P*-values, LSS performed better than HMM.

Moreover, we also compared LSS’s results with *groHMM* and *MACS2*. In this RECWT25 sample, *groHMM* only found 17 genes with a significant bin-level point, and *MACS2* only found 16, much less than LSS’s 88 (Supplementary Fig. S6A and B). Among them, 16 and 15 genes were shared with LSS, respectively.

Further comparison on the base level still confirmed the higher accuracy of LSS, because all the 88 LSS points were significant on the base level, giving a significance proportion of 1, which was higher than *groHMM*’s 0.941 and *MACS2*’s 0.875 (Supplementary Fig. S6C and D). In addition, the LSS points had smaller significance *P*-values on the base level (Supplementary Fig. S6E and F). Hence, LSS performed better than *groHMM* and *MACS2*.

In addition, we also tested the LSS, HMM, *groHMM*, and *MACS2*’s performance on the RECKD25 group, which also showed that LSS identified a significant transition point in more genes, and its points were more significant on the base level (Supplementary Figs S7 and S8).

Next, we selected 164 genes ≥40 kb with an FPKM >1 from the RECWT0 data to generate simulated datasets and explore the factors relevant to LSS’s accuracy, defined as the proportion of inferred transition points ≤50 bp from the true ones. This time, the depleted/intact FPKM ratio (the parameter Difference) and the intact region’s depth (the parameter Depth) showed a large influence (Fig. 4F). As the FPKM ratio increased from the start value of 0.02, which indicated the depleted region’s FPKM was much smaller than the intact one, to its final value of 0.75, which meant the two regions’ FPKMs were similar, the accuracy decreased sharply from 0.843 to 0.484. On the other hand, as the intact region’s depth increased from 0.25 to 2, i.e. its depth changed from 25% to 200% of the original RECWT0 file, the accuracy increased fast from 0.568 to 0.872. In addition, the depleted region’s length (the parameter Distance) and the *window_num* parameter of the function *calrate*, also influenced LSS’s accuracy, but not as obvious as the former factors. However, in most cases, the accuracy was >0.8.

Furthermore, using the simulated negative dataset from these 164 genes, we found that LSS still had excellent performance in excluding false positive transition points, because when the *window_num* parameter changed from 10 to 200, LSS achieved a false positive rate = 0 in almost all the cases (Supplementary Fig. S5).

After validating LSS’s reliability on this dataset, we used its results to explore the biological function of RecQL5. The inferred transcription rates showed that the RECKD cells were faster (Fig. 3D). Correspondingly, *calpauseidx* showed that RECKD0 cells had smaller pause indices than RECWT0, meaning Pol II could enter the gene body more efficiently in the knockdown condition. In addition, the RECKD0’s genes also had smaller FPKMs than RECWT0, which could also be seen from the results of *mmetaplot* and *plotprocessing*, showing that the RECKD0’s metagene had less nascent RNAs around the promoter (Fig. 3E). However, they had similar gene body RNAs to RECWT0 (Fig. 3F). Hence, the results of RecQL5 were opposite to the former Paf1C one. The knockout of Paf1C increased promoter accumulated RNAs, increased genes’ pause indices, and slowed their transcription. In contrast, the knockdown of RecQL5 decreased promoter accumulated RNAs, decreased pause indices, and accelerated transcription. However, the Wilcoxon *P*-values of these RecQL5 results were insignificant; one possible reason was the small gene number used in the test, and the other was that RecQL5 was just knocked down rather than being completely KO as Paf1C, making the difference from the WT cells not as obvious.

This case study showed the performance of LSS in the difficult RecQL5 dataset.

### LSS infers the mouse embryonic stem cell transcription rates more accurately than HMM

We also tested the package using the GEO dataset GSE48895 [15]. This time, mouse embryonic stem cells (mESCs) were treated with Flavopiridol (FP) for 12 min (FP12 cells) or left untreated (FP0 cells). FP had a similar effect as DRB. It also prevented Pol II from entering the gene body for transcription [16]. Then, the cells were sequenced to generate single-end Gro-seq data. After preprocessing, we kept the reads with a mapping quality score ≥10 in the bam files, and we analyzed the genes with a length ≥40 kb and an FPKM >1 in the FP0 data.

**Figure 5.**
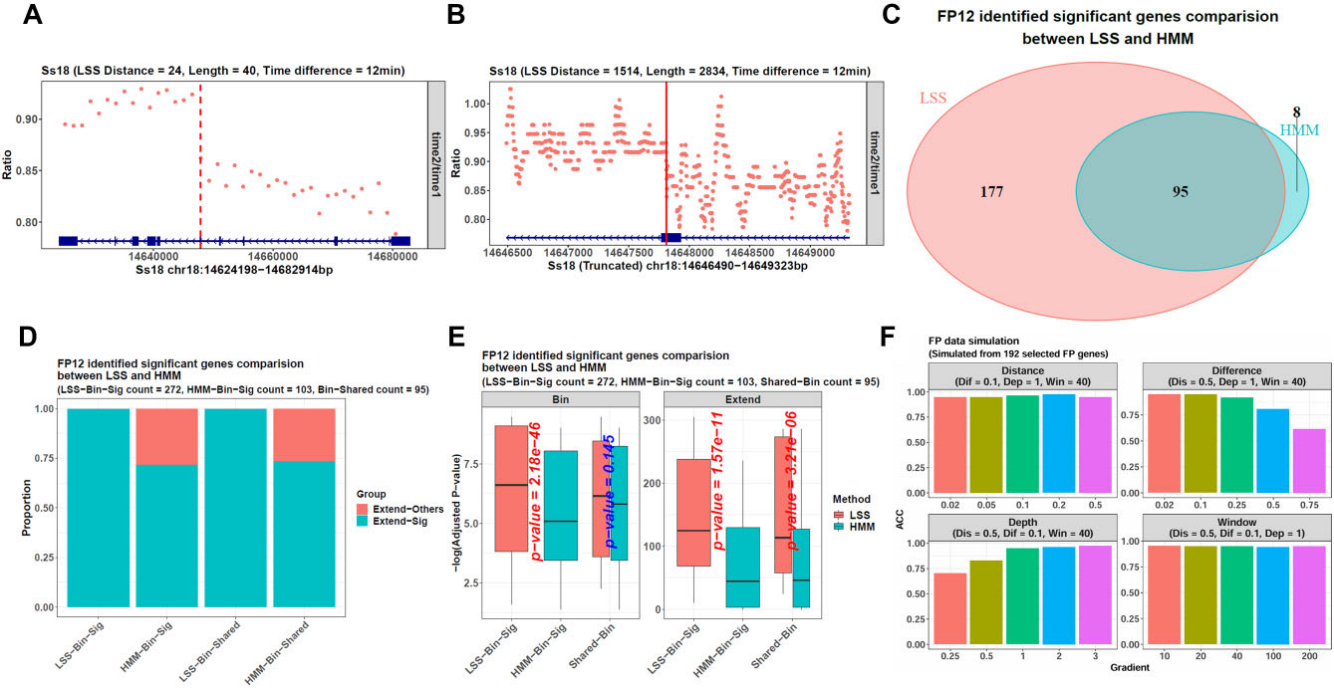
LSS infers the mESC transcription rates more accurately than HMM. (**A**) In the FP12 data, *calrate* identifies the gene Ss18’s transition point in a bin-expansion manner. It identifies the 24th bin with the LSS method on the bin level. The dots represent the normalized read count ratios between the FP12 and FP0 bins. The vertical dotted line indicates the transition bin. (**B**) On the base level, *calrate* expands the 24th and 25th bins and uses LSS in this region. The dots represent the normalized read count ratios between the FP12 and FP0 bases. It identifies the base 1514 as the final point, as labeled by the vertical solid line. This point is also the base 35 105 of the whole gene Ss18, which is 58 717-bp long. (**C**) In the FP12 data, LSS finds a significant transition point in 272 genes with a length ≥40 kb and an FPKM >1 in the reference. On the other hand, HMM identifies a significant point in 103 genes. Among them, 95 are shared by the two methods. (**D**) All the LSS and HMM points are significant on the bin level. However, on the base level, all the LSS points in the 272 genes (the LSS-Bin-Sig point group) are still significant, but the HMM points in the 103 genes (the HMM-Bin-Sig point group) only contain 74 significant ones with a significance proportion of 74/103 = 0.718. Furthermore, for the 95 shared genes, their points identified by LSS (the LSS-Bin-Shared point group) are all significant on the base level. However, the ones identified by HMM (the HMM-Bin-Shared point group) only contain 70 significant ones with a significance proportion of 70/95 = 0.737. The cyan parts represent the point groups’ significance proportions, and the red ones represent insignificance proportions. (**E**) Although all the identified points are significant on the bin level, the 272 LSS-Bin-Sig points have much smaller significance *P*-values than the 103 HMM-Bin-Sig points on both the bin and base levels (the facets Bin and Extend). For the 95 shared genes (Shared-Bin genes), their transition points identified by LSS have much smaller significance *P*-values on the base level (the facet Extend). (**F**) The simulated datasets are generated from the FP0 data and have pre-defined gene transition points. Their inference results show that LSS’s accuracy is influenced by the depleted region’s length (Distance), the intact region’s depth (Depth), the FPKM ratio between these regions (Difference), and the bin number used in the bin-expansion framework (Window). When the Distance value is fixed as 0.5, the Depth value is fixed as 1, and the Window value is fixed as 40 (the top-right facet), i.e. the depleted region’s length is 50% of the corresponding gene, the intact region’s sequencing depth is the same as the original FP0 data, and the bin number used in the bin-expansion framework is 40, LSS’s accuracy decreases sharply from 0.948 to 0.615 as the Difference parameter increases from 0.02 to 0.75, i.e. as the depleted region’s FPKM increases from 2% to 75% of the intact one. On the other hand, the parameter Depth also shows an effect because, in the condition of Distance = 0.5, Difference = 0.1, and Window = 40 (the bottom-left facet), LSS’s accuracy increases from 0.703 to 0.974 as the intact region’s depth increases from 25% to 300% of the original FP0 (from Depth = 0.25 to Depth = 3). In addition, the parameters Distance and Window can also influence LSS’s accuracy independently when fixing other parameters (the top-left and bottom-right facets).

This time, LSS identified a significant transition point in 272 genes, such as Ss18, which was 58 717-bp long. LSS found its 24th bin as the transition bin (Fig. 5A), and the base 1514 in the expanded bin region was the final point (Fig. 5B), which was also the base 35 105 of the whole gene.

In addition to the 272 genes from LSS, HMM found a significant point in 103 genes, still less than LSS. Among them, 95 genes were shared by both methods (Fig. 5C). In most cases, the points identified by LSS and HMM were different, even if they were in the same gene. Furthermore, the comparison on base-level significance confirmed the higher accuracy of LSS because although all 272 LSS points were significant, only 74 of the 103 HMM points were base-level significant. Hence, HMM’s significance proportion was only 74/103 = 0.718, less than that of LSS as 1 (Fig. 5D). Meanwhile, the LSS points also had smaller significance *P*-values on both the bin and base levels (Fig. 5E). Hence, the conclusion here was the same as the former case studies that LSS performed better than HMM.

In addition, LSS also showed a large advantage over *groHMM* and *MACS2*. In this FP12 sample, *groHMM* only found 29 genes with a significant bin-level point, and *MACS2* only found 53 genes, much less than LSS’s 272 (Supplementary Fig. S9A and B). Among them, 27 and 51 genes were shared with LSS, respectively.

Further comparison on the base level still confirmed the higher accuracy of LSS, because all the LSS points, but not all the *groHMM* and *MACS2* points, were significant on the base level. Their significance proportions were 1 for LSS, 0.966 for *groHMM*, and 0.887 for *MACS2* (Supplementary Fig. S9C and D). In addition, the LSS points had smaller significance *P*-values on the base level (Supplementary Fig. S9E and F). Hence, LSS performed better than *groHMM* and *MACS2*.

Then, further analysis confirmed the relationship between LSS’s accuracy and the four factors: the depleted region’s length (the parameter Distance), the intact region’s depth (the parameter Depth), the FPKM ratio between them (the parameter Difference), and the parameter *window_num* of the function *calrate*. This time, we selected 192 genes ≥40 kb with an FPKM >1 from the FP0 data to construct different simulated datasets with pre-defined transition points. The depleted/intact FPKM ratio still showed an obvious influence on LSS, and as its value increased from 0.02 to 0.75, LSS’s accuracy decreased largely from 0.948 to 0.615 (Fig. 5F). In addition, as the intact region’s sequencing depth increased from 0.25 to 3, the accuracy increased from 0.703 to 0.974. Besides, the parameters Distance and *window_num* also influenced LSS’s accuracy, but not as obvious as the former Difference and Depth. Hence, the conclusion here was the same as before: LSS achieved a higher accuracy in the condition of a large depleted/intact FPKM difference, a large intact region’s depth, a longer depleted region’s length, and a *window_num* value of 40.

Furthermore, the genes’ simulated negative dataset confirmed LSS’s strong ability to exclude false positive points, because as the *window_num* parameter changed from 10 to 100, the false positive rate was consistently 0. Only when *window_num* became 200, it increased slightly to 0.027 (Supplementary Fig. S5).

It was noteworthy that, although the simulated datasets for the Paf1C, RecQL5, and mESC case studies only contained 150, 164, and 192 genes, respectively, the consistency of their results showed the reliability of the conclusion.

**Figure 6.**
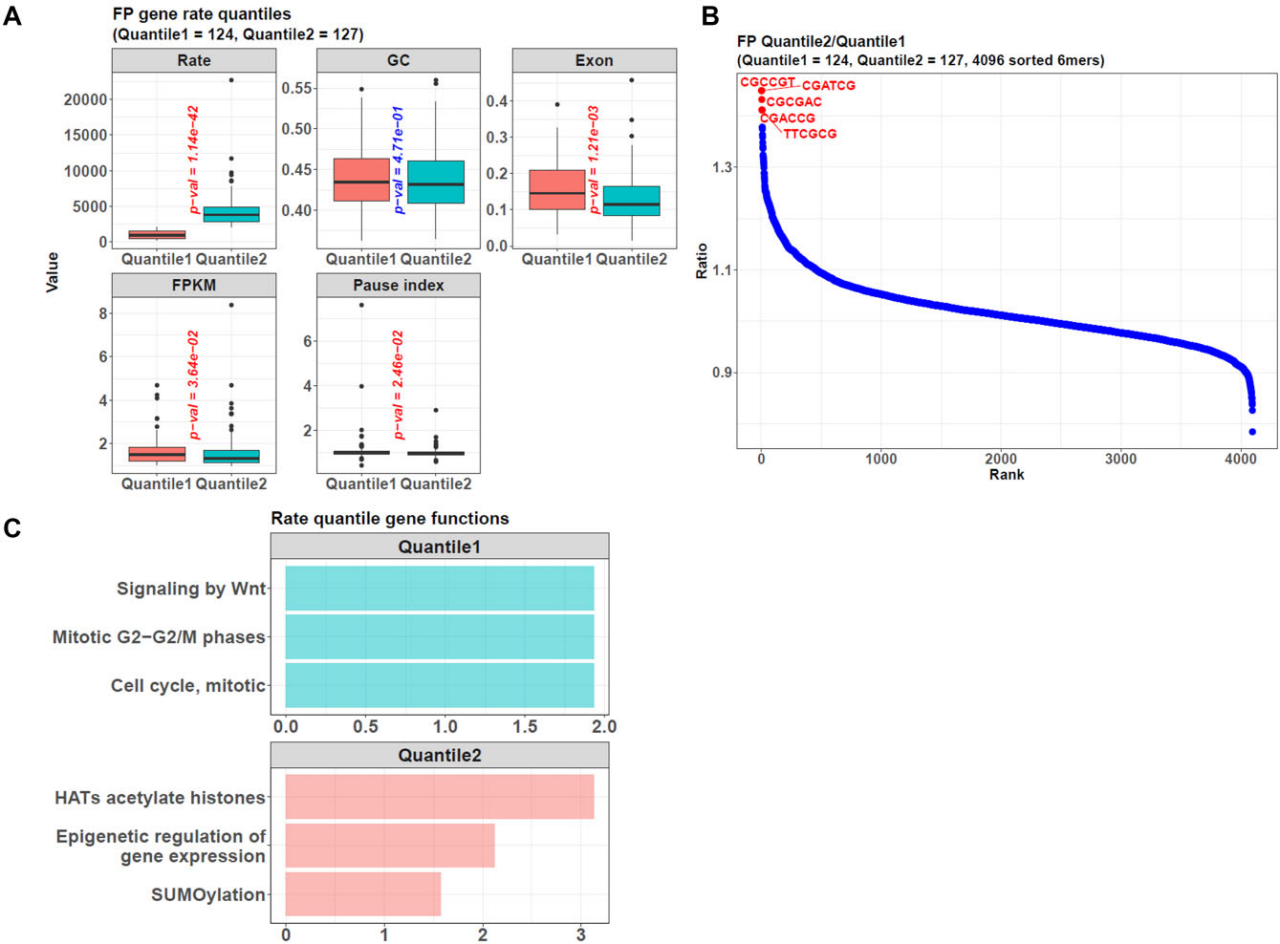
The package detects the structural and functional differences between faster and slower transcribed genes. (**A**) From the result of *calrate* on the mESC dataset, the genes can be divided into two clusters according to the 50% quantile of all inferred rates from LSS. The Quantile1 cluster includes genes with a rate less than that value, and the Quantile2 cluster includes genes with a larger rate. In addition, the two clusters also show other differences because the faster genes in Quantile2 have smaller GC contents, exon densities, FPKM values, and pause indices, hinting that genes with fewer GC base pairs and fewer exons tend to be transcribed faster, and their Pol II can enter the gene bodies more efficiently. All the results are based on genes with a length ≥40 kb and an FPKM >1 in the reference cells. Furthermore, because a gene’s pause index is calculated as the ratio of the Pol II density 1 kb around its TSS to its gene body, it will be excluded if this gene overlaps with others in these regions. (**B**) From the results of *getkmer*, the Quantile2 genes are positively enriched in 6-mer sequences such as CGCCGT, CGATCG, and CGCGAC. Each dot represents a 6-mer, and its value on the *y*-axis represents the 6-mer’s frequency ratio between the Quantile2 and Quantile1 gene sequences. Along the *x*-axis, all the dots are ordered according to their ratios on the *y*-axis. (**C**) Gene set functional enrichment shows that the slower genes in Quantile1 are enriched in the “Signaling by Wnt” and “Cell cycle, mitotic” functions. On the other hand, the faster genes in Quantile2 are closely related to functions of “Epigenetic regulation of gene expression” and “SUMOylation”. The *x*-axis represents −log_10_(adjusted *P*-value) for the enrichment.

Finally, we explored the biological factors influencing the transcription rates in this mESC dataset. We divided the genes into two clusters according to the 50% quantile of all inferred rates from LSS. It was natural that the gene cluster with rates less than that value (the Quantile1 cluster) was significantly slower than the other cluster with greater rates (the Quantile2 cluster) (Fig. 6A). Correspondingly, the Quantile2 genes’ FPKM values and pause indices were smaller than the Quantile1 genes’, as shown by the results of *calrate* and *calpauseidx*. Hence, the conclusion here was similar to the former case studies that faster genes’ Pol II tended to enter gene bodies rather than accumulate at promoter regions so that transcription could be conducted more efficiently.

In addition, we compared the two clusters’ gene GC (guanine-cytosine) contents and exon densities, which were returned by *calrate* or could be calculated by the functions *getgc* and *getexon* in our package. The result showed that the faster genes in Quantile2 had significantly smaller exon densities, and their GC contents were also smaller.

However, this contradicted some reports about nuclear speckles, a subregion of efficient transcription, and mRNA processing in the nucleus [17–19]. It was found that many active genes reproducibly position near nuclear speckles, and the speckle-localized pre-mRNAs were relatively short, GC-rich, and exhibited a preference for strong 5’ and 3’ splice sites with a high exonic machine learning score. Hence, the active genes here had a high GC content, rather than a low one as concluded by our transcription rate analysis. This inconsistency was because the speckle-localized pre-mRNAs not only had high transcript levels, but also underwent efficient splicing, and their high GC contents mainly contributed to the splicing [17, 20]. However, from the transcription aspect, the high GC contents were not a favorable factor. To overcome this and still achieve a high transcript level, the nascent RNAs in nuclear speckles were coupled with reduced exosome RNA degradation and larger Ser2p C-terminal domain-modified RNA Pol II foci, as demonstrated by some initial investigations [19].

In addition to the GC content study above, we also checked the detailed sequences of the faster and slower genes. Using the function *getkmer* in our package, we compared the clusters Quantile1 and Quantile2’s sequences and found that the faster Quantile2 genes were positively enriched in 6-mer sequences such as CGCCGT, CGATCG, and CGCGAC (Fig. 6B).

Finally, gene set functional enrichment also showed that the two clusters differed (Fig. 6C). The slower genes in Quantile1 were in charge of functions such as “Signaling by Wnt” and “Cell cycle, mitotic”. In contrast, the faster genes in Quantile2 were enriched in “Epigenetic regulation of gene expression” and “SUMOylation”, which tended to change quickly to respond to environmental stimulations.

This was consistent with the observation that genes tended to have faster transcription rates if they needed to be rapidly adjusted in response to an environmental cue, such as the histone genes [21, 22]. Their RNA amounts were the results of their high transcription rates that were compensated by their severe mRNA instabilities. These genes followed a strategy with very high transcription rates but very low mRNA stability that allowed for fast changes in RNA amounts [23, 24]. Thus, epigenetic regulatory genes, such as the histone ones, were among the highest transcribed genes.

This case study showed the performance of our package in inferring mESC cells’ transcription rates and analyzing corresponding gene structures and functions.

## Discussion

Transcriptional elongation is linked to various biological activities, including RNA splicing, polyadenylation, nuclear export, etc. [1–5], and a mutant change in transcription rates can further lead to defects in these processes [6, 7]. Hence, inferring elongation rates becomes an important task in these dynamic studies. Traditionally, the HMM model is used to achieve this from Pro-seq or Gro-seq data [8, 9]. Here, we developed the R package *proRate*, which uses a novel LSS method instead.

The essence of this task is to identify the transition point between a gene’s read-depleted region and intact region, which is formed by using drugs, such as DRB, to prevent Pol II from entering the gene body. In this case, HMM needs to (i) estimate its model’s various parameters and (ii) use the model to infer the regions’ depleted/intact states from the observed read ratios. As long as the model is determined, the second step for state inference can be completed with Viterbi decoding, a special case of the belief propagation algorithm. However, it is more difficult to determine the model at the first step because (i) to solve its parameters with the maximum likelihood estimation (MLE) method, the regions’ depleted/intact states should be known, but they are not; (ii) the observed read ratios are from continuous distributions, whose family is difficult to determine but necessary to MLE. For the second problem, it is typical to assume the ratios are from normal distributions. For the first problem, the unknown regions’ states can be treated as a hidden variable. Then, it can be estimated with other parameters simultaneously, which is achieved via the EM iteration, a special case of MLE, and in HMM, it is called the Baum–Welch algorithm [10]. However, the assumption that the read ratios follow normal distributions cannot always be true. Also, because of the introduction of a hidden variable, the model parameters cannot be estimated accurately, which impairs HMM’s performance in the transition point identification.

Hence, we tried to overcome these defects and finally found our new method. It has no assumptions on the ratios’ distribution family, so the concern about the normal distributions’ fitness can be avoided. More importantly, it eliminates the parameter estimation, which is based on MLE in HMM and is the main source of HMM’s limitations. Instead, we calculate the read ratios’ variances, which do not depend on any model’s parameter estimation, and use the variances’ SS value to identify the regions’ states, so we call this method LSS.

Notably, our LSS method can be seen as a special case of *k*-means clustering, where *k* = 2. It treats the depleted and intact regions as two clusters and divides the gene base pairs into them. The original *k*-means method determines the optimal clustering from various candidates, where some base pairs are combined and attributed to the first cluster, and others are assigned to the second one. For a candidate cluster, it also calculates its read ratios’ variance around their mean. Then, it sums up the two clusters’ variances in the candidate clustering to get the SS value, and finally, the optimal clustering is the one with the smallest SS. Hence, both the original *k*-means and our method select the optimal clusters via SS. However, the two methods’ clustering candidates are different. In *k*-means, a cluster can contain any base pair combination without considering whether they are neighbors or not, so for a gene with *n* base pairs, it has 0.5 × 2*^n^* clustering candidates to find the optimal. On the contrary, our method constrains that the base pairs in the same cluster should be neighbors naturally because a gene only has one continuous depleted region and one continuous intact region. Hence, the same gene only has *n* clustering candidates, much less than the *k*-means’ 0.5 × 2*^n^*. Furthermore, to process the large candidate number within a limited time, *k*-means has to use a greedy algorithm, ignoring many candidates and finally returning an approximate solution. In contrast, all the *n* candidates in our method can be evaluated by dividing the gene at each base pair, so the final solution is exact out of all its candidates.

Hence, our LSS method has advantage over both HMM and *k*-means, mainly because of its constraint on the regions’ continuity. However, this also means LSS cannot be used in solving more complicated problems with interrupted regions or more than two region states, such as determining the chromosome open/close states, where the open/close regions are interrupted and distributed across the genome, rather than linking into two continuous regions. In this case, HMM can still be used to solve it [25, 26], but LSS cannot.

In summary, we developed the package *proRate*. It aims to infer transcription rates from DRB-based Pro-seq or Gro-seq data and performs more accurately than HMM. In addition, it offers other functions, such as metagene identification, pause index calculation, and gene structure analysis, which are frequently used in transcription dynamic studies. Hence, *proRate* can be very helpful in this area.

## Supplementary Material

lqaf123_Supplemental_File

## Data Availability

*proRate* is available on GitHub (https://github.com/yuabrahamliu/proRate) and Zenodo (https://doi.org/10.5281/zenodo.16780395).
