# Supplementary material for: proRate: an R package to infer gene transcription rates with a novel least sum of squares method
Source: NAR Genom Bioinform. 2025 Sep 5;7(3):lqaf123. doi: 10.1093/nargab/lqaf123 (PMC12412782; doi:10.1093/nargab/lqaf123)
Supplement: lqaf123_Supplemental_File [file lqaf123_supplemental_file.pdf]

Supplementary Data

Contents

Supplementary Figures **2**

Supplementary Methods **21**

Data collection and preprocessing 21

Transcription rates inference 21

Transcription rates inference with *groHMM* and *MACS2* 23

Simulated data generation 24

Pause index calculation 25

Metagene and gene structure analyses 26

Gene functional enrichment analysis 27

References **28**

Supplementary Figures

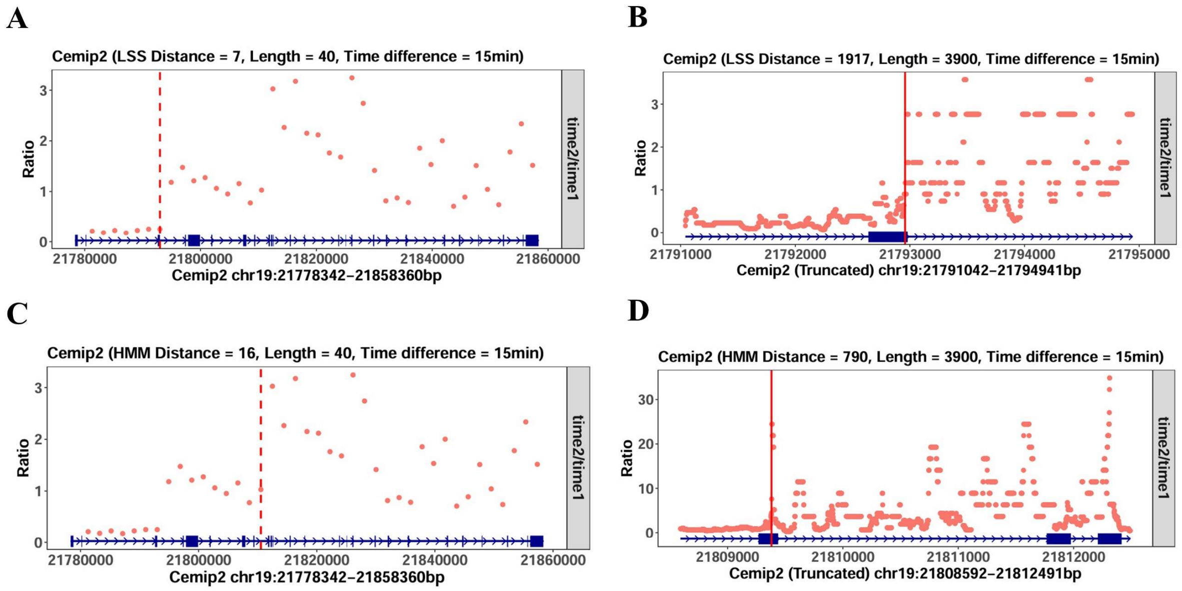

Figure S1. LSS and HMM identify different transition points in the Cemip2 gene in mouse C2C12 cells. (A) In the WT15 data, the LSS method of *calrate* identifies the gene Cemip2's depleted/intact transition point in a bin-expansion framework. On the bin level, it identifies the 7th bin with the LSS method. (B) On the base level, *calrate* expands the 7th and 8th bins and uses LSS in this region. It identifies the base 1917 as the final point. This point is the base 14617 of the whole gene Cemip2, whose length is 80019 bp. (C) The HMM method of *calrate* identifies the 16th bin as Cemip2's transition bin. (D) On the base level, *calrate* expands the 16th and 17th bins and uses HMM in this region. It identifies the base 790 as the final point, which is also the base 31040 of the whole gene Cemip2. In (A) and (C), the dots represent the normalized read count ratios between the WT15 and WT0 bins, and the vertical dotted line labels the identified bin. In (B) and (D), the dots represent the normalized read count ratios

between the WT15 and WT0 bases, and the vertical solid line indicates the final point identified.

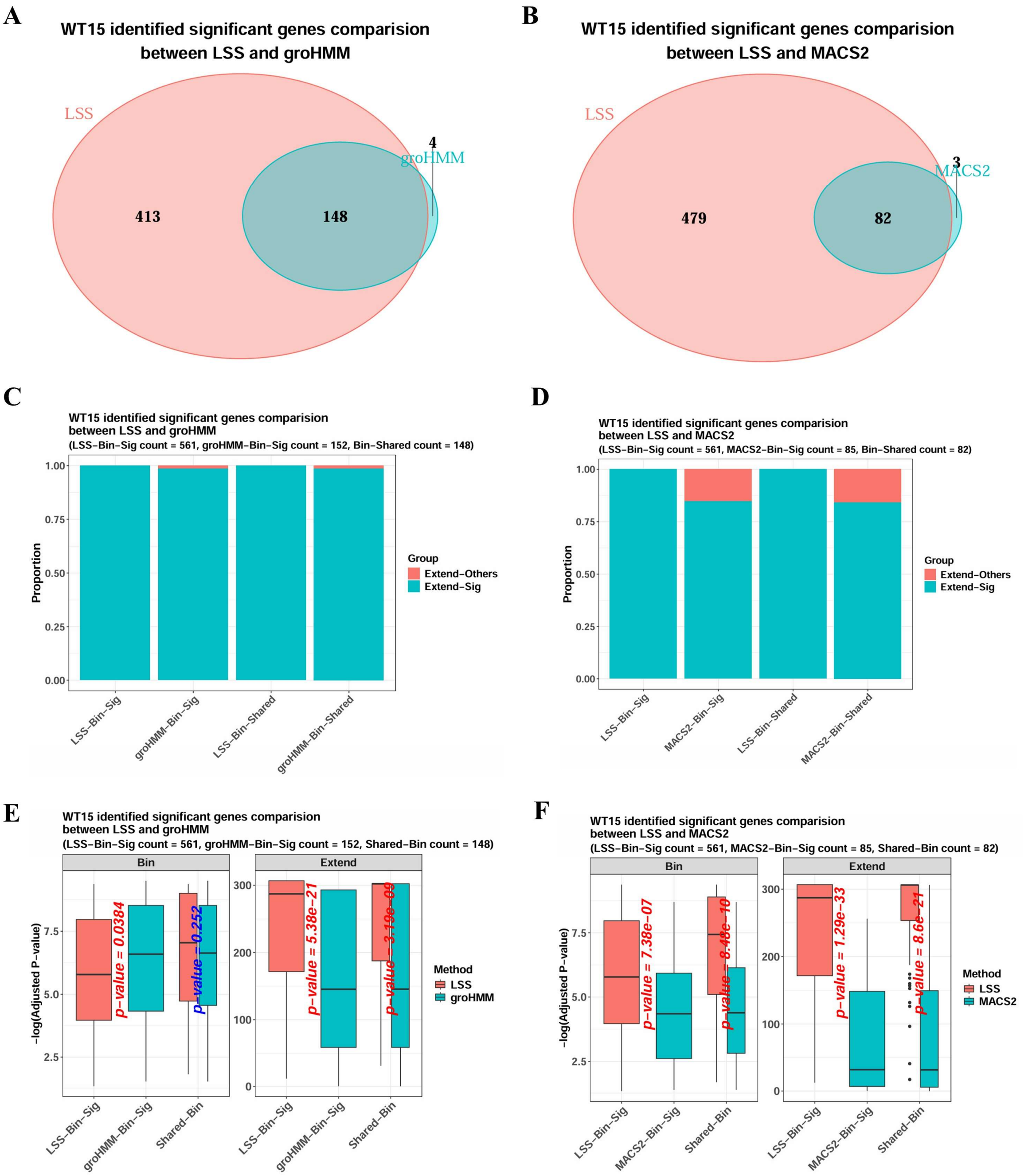

Figure S2. LSS infers the mouse C2C12 cell transcription rates more accurately than *groHMM* and *MACS2*. (A) In the WT15 data, LSS finds a significant transition point in 561 genes with a length >= 40 kb and an FPKM > 1 in the WT0 reference. However, *groHMM* only identifies a significant point in 152 genes. Among them, 148 genes are shared. (B) On the other hand, *MACS2* identifies a significant point in 85 genes with 82 of them shared with LSS. (C)

All the LSS and *groHMM* identified transition points are significant on the bin level. However, if checking them in the expanded regions on the base level, the *groHMM* points in the 152 genes (the *groHMM*-Bin-Sig point group) contain 150 significant ones, giving a significance proportion as high as 150/152 = 0.987, which is still less than LSS because all the LSS points in the 561 genes (the LSS-Bin-Sig point group) are significant, giving a significance proportion as 1. Furthermore, for the 148 shared genes, their points identified by LSS (the LSS-Bin-Shared point group) are all significant on the base level. On the other hand, the ones identified by *groHMM* (the *groHMM*-Bin-Shared point group) contain 146 significant ones with a significance proportion of 146/148 = 0.986. (D) In addition, all the *MACS2*-identified transition points are also significant on the bin level. However, on the base level, the 85 *MACS2* points (the *MACS2*-Bin-Sig point group) contain 72 significant ones, giving a significance proportion of 72/85 = 0.847, which is less than LSS's significance proportion of 1. Furthermore, for the 82 shared genes, their points identified by *MACS2* (the *MACS2*-Bin-Shared point group) contain 69 significant ones with a significance proportion of 69/82 = 0.841. In (C) and (D), the cyan parts represent the point groups' significance proportions, and the red ones represent insignificance proportions. (E) On the bin level (the facet Bin), the 152 *groHMM*-Bin-Sig points have smaller significance p-values than the 561 LSS-Bin-Sig points. In contrast, on the base level (the facet Extend), the LSS-Bin-Sig points have smaller significance p-values than the *groHMM*-Bin-Sig points. If checking the 148 shared genes (Shared-Bin genes) on the base level (the facet Extend), their transition points identified by LSS also have smaller significance p-values than those identified by *groHMM*. (F) The 561 LSS-Bin-Sig points have smaller significance p-values than *MACS2* on both the bin and base levels (the facets Bin and Extend).

Moreover, for the 82 shared genes of LSS and *MACS2* (Shared-Bin genes), their transition points identified by LSS also have smaller p-values than *MACS2*, on both the bin and base levels.

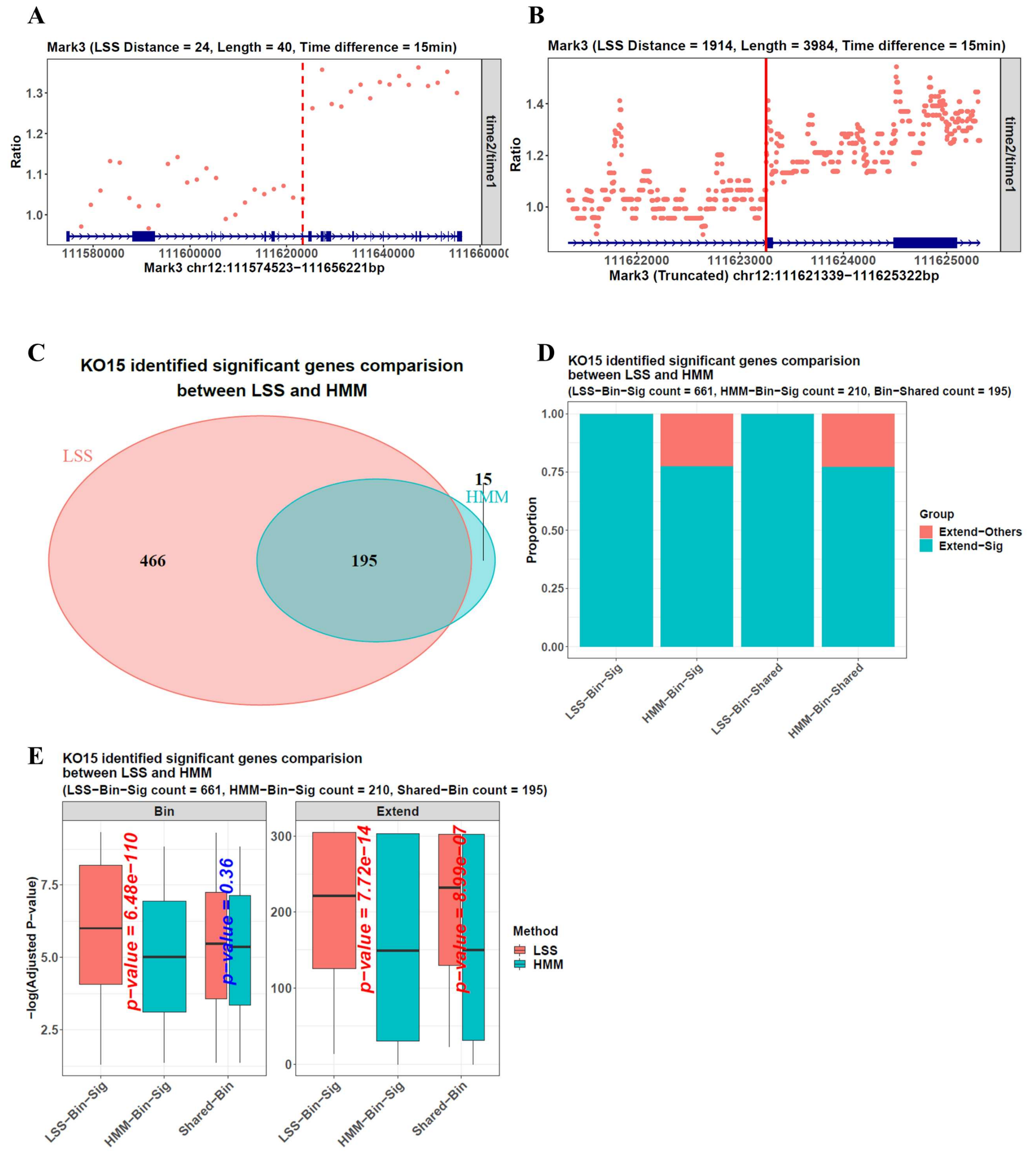

Figure S3. LSS infers the Paf1C knockout gene transcription rates more accurately than HMM. (A) In the KO15 data, *calrate* identifies the gene Mark3's depleted/intact transition point in a bin-expansion framework. It identifies the 24th bin with the LSS method on the bin level. The dots represent the normalized read count ratios between the KO15 and KO0 bins. The vertical dotted line labels the identified bin. (B) On the base level, *calrate* expands the 24th

and 25th bins and uses LSS in this region. The dots represent the normalized read count ratios between the KO15 and KO0 bases. It identifies the base 1914 in this region, as labeled by the vertical solid line. This point is also the base 48730 of the whole gene Mark3, whose length is 81699 bp. (C) In the KO15 data, LSS finds a significant transition point in 661 genes with a length >= 40 kb and an FPKM > 1 in the KO0 reference. However, HMM only identifies a significant point in 210 genes. Among them, 195 genes are shared. (D) All the identified transition points are significant on the bin level. However, if checking them in the expanded regions on the base level, all the LSS points in the 661 genes (the LSS-Bin-Sig point group) are significant, but the HMM points in the 210 genes (the HMM-Bin-Sig point group) only contain 163 significant ones. Hence, their significance proportion is 163/210 = 0.776. Furthermore, for the 195 shared genes, their points identified by LSS (the LSS-Bin-Shared point group) are all significant on the base level. However, the ones identified by HMM (the HMM-Bin-Shared point group) only contain 151 significant ones with a significance proportion of 151/195 = 0.774. The cyan parts represent the point groups' significance proportions, and the red ones represent insignificance proportions. (E) On both the bin and base levels (the facets Bin and Extend), the 661 LSS-Bin-Sig points have much smaller significance p-values than the 210 HMM-Bin-Sig points. If checking the 195 shared genes (Shared-Bin genes) on the base level (the facet Extend), their transition points identified by LSS also have smaller significance p-values than those identified by HMM.

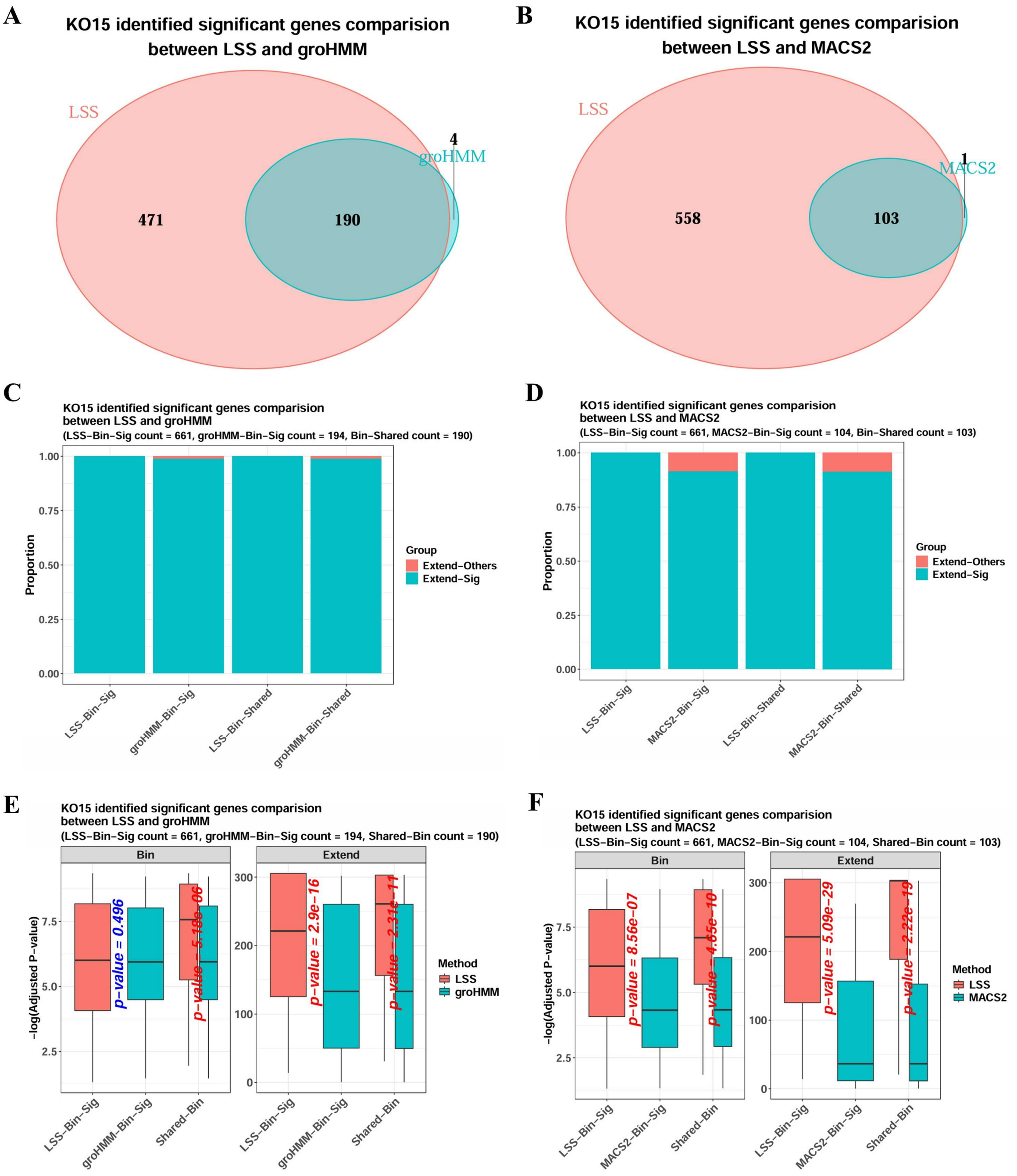

Figure S4. LSS infers the Paf1C knockout gene transcription rates more accurately than *groHMM* and *MACS2*. (A) In the KO15 data, LSS finds a significant transition point in 661 genes with a length >= 40 kb and an FPKM > 1 in the KO0 reference. However, *groHMM* only identifies a significant point in 194 genes. Among them, 190 genes are shared. (B) On the other hand, *MACS2* identifies a significant point in 104 genes, with 103 genes shared with LSS. (C)

All the LSS and *groHMM* identified transition points are significant on the bin level. However, if checking them in the expanded regions on the base level, all the LSS points in the 661 genes (the LSS-Bin-Sig point group) are significant, whereas the *groHMM* points in the 194 genes (the *groHMM*-Bin-Sig point group) contain 192 significant ones. Hence, their significance proportion is 192/194 = 0.99. Furthermore, for the 190 shared genes, their points identified by LSS (the LSS-Bin-Shared point group) are all significant on the base level, whereas the ones identified by *groHMM* (the *groHMM*-Bin-Shared point group) contain 188 significant ones with a significance proportion of 188/190 = 0.99. (D) In addition, all the *MACS2*-identified transition points are significant on the bin level. However, on the base level, these 104 points (the *MACS2*-Bin-Sig point group) contain 95 significant ones. Hence, their significance proportion is 95/104 = 0.913. Moreover, for the 103 shared genes, their points identified by *MACS2* (the *MACS2*-Bin-Shared point group) contain 94 significant ones with a significance proportion of 94/103 = 0.913. In (C) and (D), the cyan parts represent the point groups' significance proportions, and the red ones represent insignificance proportions. (E) On the base level (the facet Extend), the 661 LSS-Bin-Sig points have much smaller significance p-values than the 194 *groHMM*-Bin-Sig points. If checking the 190 shared genes (Shared-Bin genes), their points identified by LSS have smaller significance p-values than *groHMM*, on both the bin and base levels (the facets Bin and Extend). (F) In addition, the 661 LSS-Bin-Sig points have smaller significance p-values than the 104 *MACS2*-Bin-Sig points on both the bin and base levels (the facets Bin and Extend), and in their 103 shared genes (Shared-Bin genes), the points identified by LSS also have smaller significance p-values than *MACS2*, on both the bin and base levels.

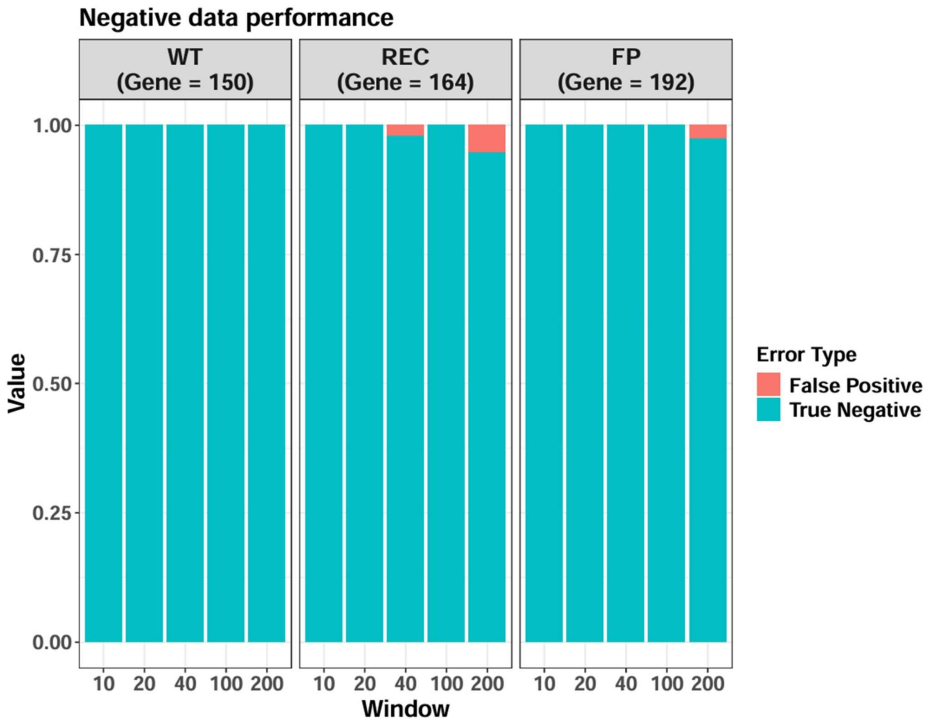

Figure S5. LSS achieves excellent performance in excluding false positive transition points. The simulated negative datasets are generated from the WT0, RECWT0, and FP0 data, respectively, with no transition points in the genes. Their inference results show that LSS achieves a false positive rate of 0 in almost all the cases. The only three exceptions appear when inferring the REC simulated data with a *window_num* parameter as 40 and 200 (false positive rate = 0.021 and 0.052) and inferring the FP simulated data with a *window_num* parameter as 200 (false positive rate = 0.027). However, these exception false positive rates are still very low, indicating LSS's strong ability to exclude false positive points. The x-axes of the plots show the different values of *window_num* used for the LSS method, and the y-axes show the false positive rate and true negative rate values.

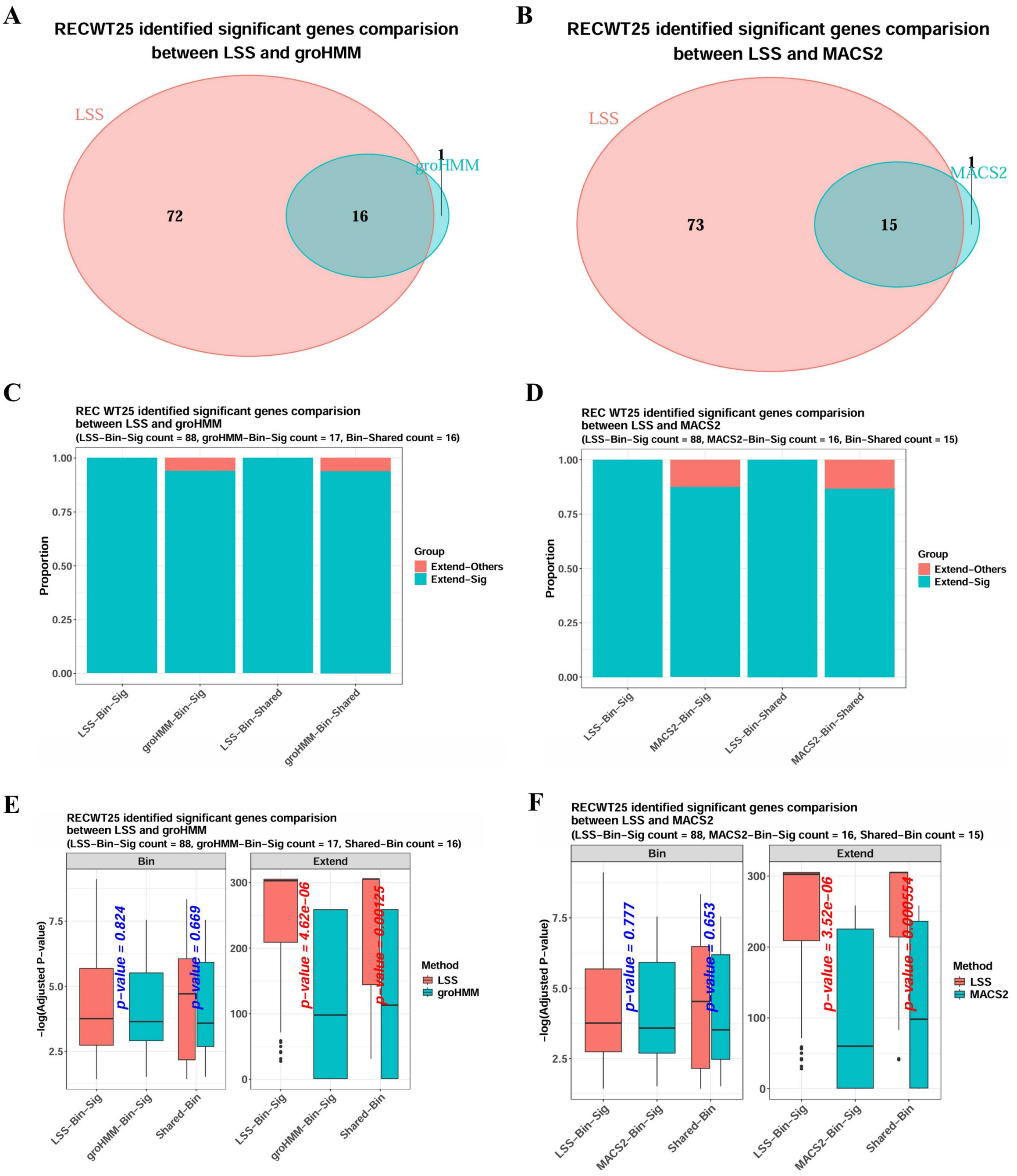

Figure S6. LSS infers the human HEK293T-Rex cell transcription rates more accurately than *groHMM* and *MACS2*. (A) In the RECWT25 data, LSS finds a significant transition point in 88 genes with a length >= 40 kb and an FPKM > 1 in the RECWT0 reference. However, *groHMM* only identifies a significant point in 17 genes. Among them, 16 genes are shared. (B) On the other hand, *MACS2* only identifies a significant point in 16 genes with 15 shared with

LSS. (C) All the LSS and *groHMM* identified transition points are significant on the bin level. However, if checking them on the base level, although the *groHMM* points have a high significance proportion of 0.941, this performance is still weaker than LSS because LSS's significance proportion is 1. Furthermore, for the shared genes, their points identified by LSS (the LSS-Bin-Shared point group) are all significant on the base level. On the other hand, the ones identified by *groHMM* (the *groHMM*-Bin-Shared point group) have a significance proportion of 0.938. (D) All the *MACS2*-identified transition points are significant on the bin level. However, on the base level, *MACS2*'s significance proportion is 0.875, less than LSS's significance proportion of 1. In addition, for the shared genes, their points identified by *MACS2* (the *MACS2*-Bin-Shared point group) have a significance proportion of 0.867. In (C) and (D), the cyan parts represent the point groups' significance proportions, and the red ones represent insignificance proportions. (E) On the bin level (the facet Bin), the 17 *groHMM*-Bin-Sig points and the 88 LSS-Bin-Sig points have similar significance p-values. However, on the base level (the facet Extend), the LSS-Bin-Sig points have smaller significance p-values than the *groHMM*-Bin-Sig points. If checking the shared genes (Shared-Bin genes) on the base level (the facet Extend), the LSS points also have smaller significance p-values. (F) On the bin level (the facet Bin), the LSS points have similar significance p-values to *MACS2*. In contrast, their significance p-values are smaller than *MACS2* on the base level (the facet Extend).

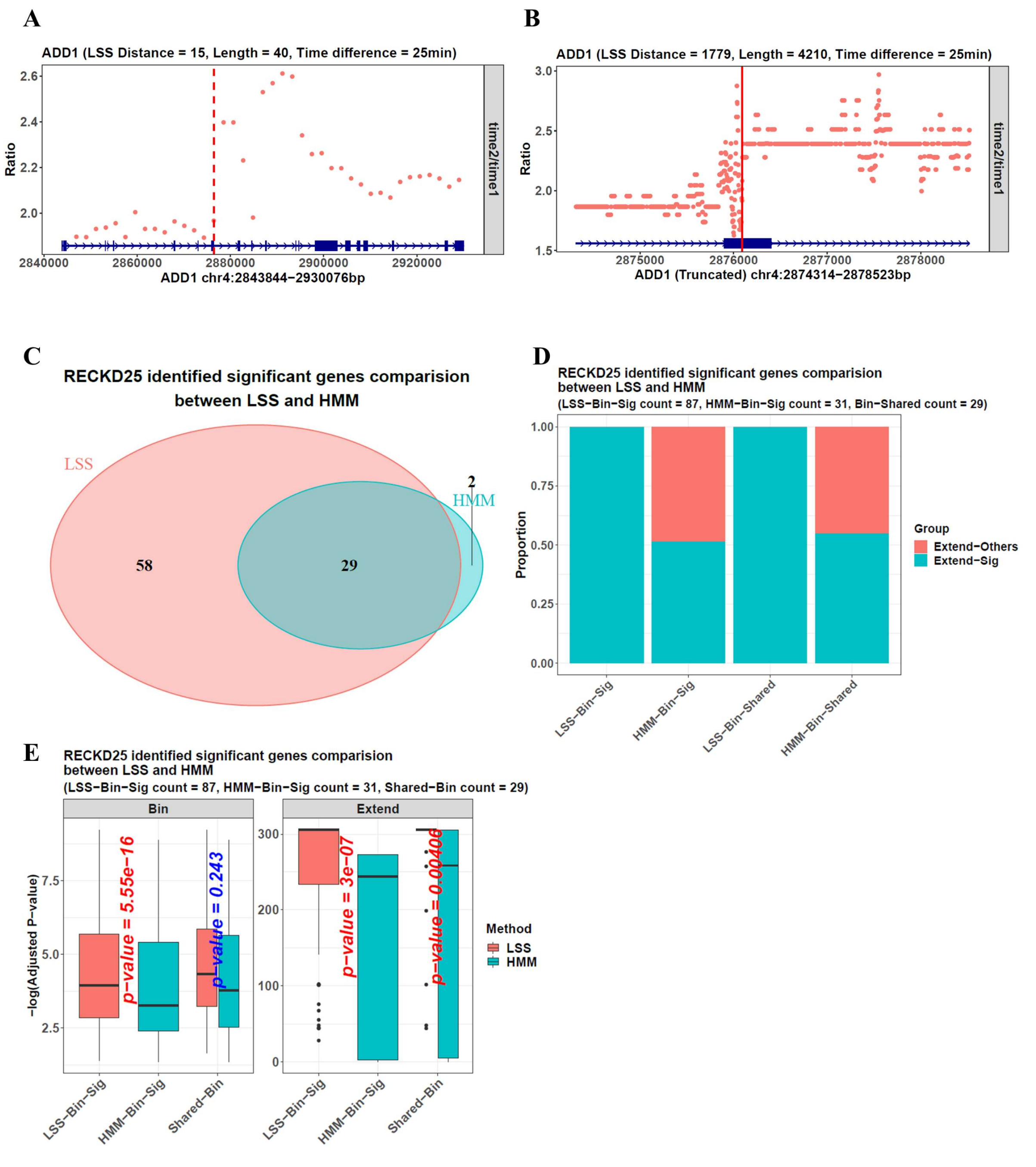

Figure S7. LSS infers the RecQL5 knockdown gene transcription rates more accurately than HMM. (A) In the RECKD25 data, *calrate* identifies the gene ADD1's transition point in a bin-expansion manner. It identifies the 15th bin with the LSS method on the bin level. The dots represent the normalized read count ratios between the RECKD25 and RECKD0 bins. The vertical dotted line labels the identified bin. (B) On the base level, *calrate* expands the 15th and

16th bins and uses LSS in this region. The dots represent the normalized read count ratios between the RECKD25 and RECKD0 bases. It identifies the base 1779 in this region, as labeled by the vertical solid line. This point is also the base 32249 of the whole gene ADD1, which is 86233 bp long. (C) In the RECKD25 data, LSS finds a significant transition point in 87 genes with a length >= 40 kb and an FPKM > 1 in the RECKD0 reference. However, HMM only identifies a significant point in 31 genes. Among them, 29 genes are shared. (D) All the identified points are significant on the bin level. However, if checking them in the expanded regions on the base level, all the LSS points in the 87 genes (the LSS-Bin-Sig point group) are significant, but the HMM points in the 31 genes (the HMM-Bin-Sig point group) only contain 16 significant ones. Hence, its significance proportion is 16/31 = 0.516. Moreover, for the 29 shared genes, their points identified by LSS (the LSS-Bin-Shared point group) are all significant on the base level. However, the ones identified by HMM (the HMM-Bin-Shared point group) only contain 16 significant ones with a significance proportion of 16/29 = 0.552. The cyan parts represent the point groups' significance proportions, and the red ones represent insignificance proportions. (E) On both the bin and base levels (the facets Bin and Extend), the 87 LSS-Bin-Sig points have much smaller significance p-values than the 31 HMM-Bin-Sig points. If checking the 29 shared genes (Shared-Bin genes) on the base level (the facet Extend), their transition points identified by LSS also have smaller significance p-values than those identified by HMM.

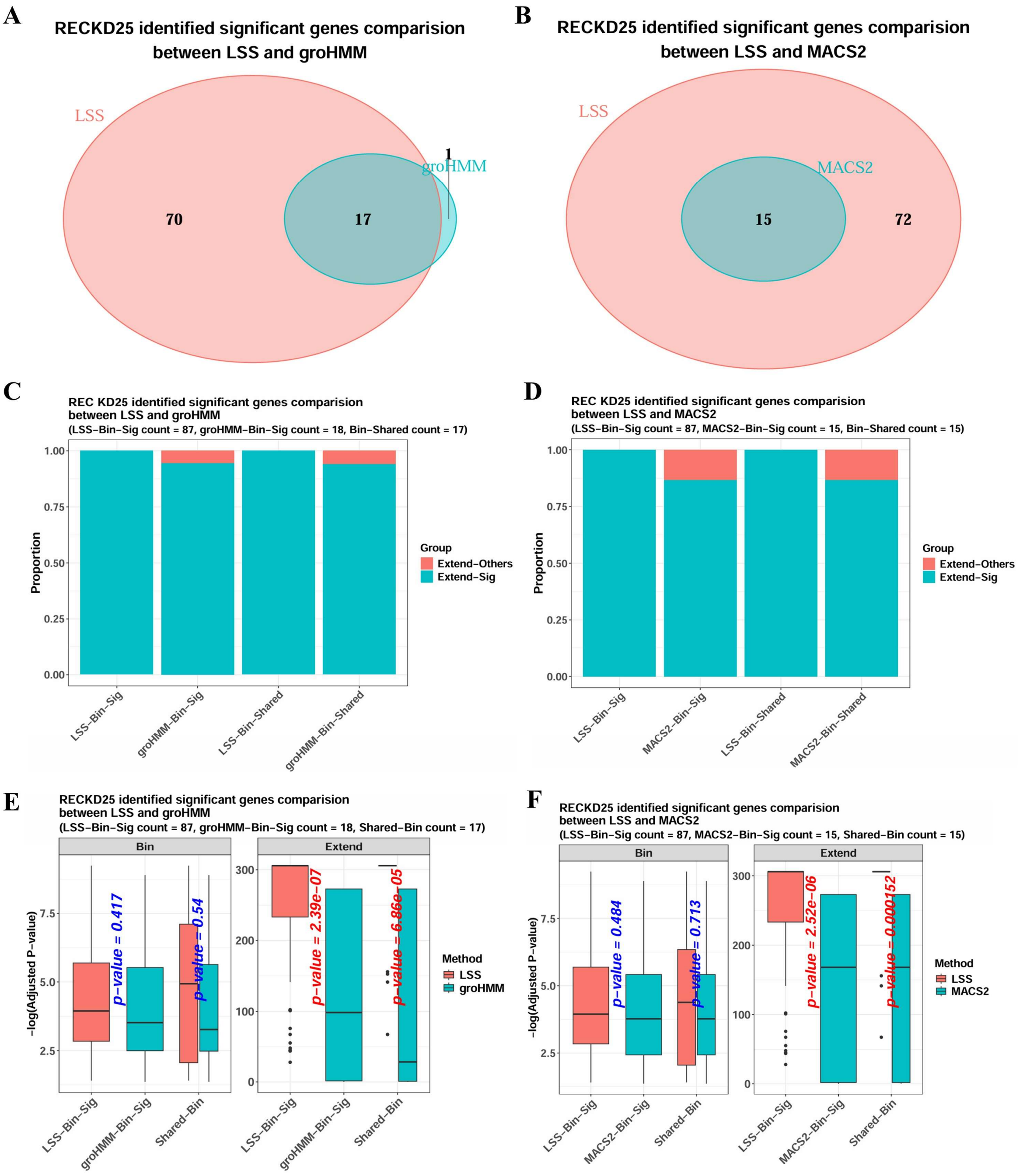

Figure S8. LSS infers the RecQL5 knockdown gene transcription rates more accurately than *groHMM* and *MACS2*. (A) In the RECKD25 data, LSS finds a significant transition point in 87 genes with a length >= 40 kb and an FPKM > 1 in the RECKD0 reference. However, *groHMM* only identifies a significant point in 18 genes. Among them, 17 genes are shared. (B) On the other hand, *MACS2* only identifies a significant point in 15 genes with all of them shared

with LSS. (C) All the LSS and *groHMM* identified transition points are significant on the bin level. However, if checking them on the base level, all the LSS points in the 87 genes (the LSS-Bin-Sig point group) are significant, whereas the 18 *groHMM* points (the *groHMM*-Bin-Sig point group) have a significance proportion of 0.944. Furthermore, for the shared genes, their points identified by LSS (the LSS-Bin-Shared point group) are all significant on the base level, whereas the ones identified by *groHMM* (the *groHMM*-Bin-Shared point group) have a significance proportion of 0.941. (D) All the 15 *MACS2* identified transition points are significant on the bin level. However, on the base level, they have a significance proportion of 0.867 (the *MACS2*-Bin-Sig point group). Because all of these 15 genes are also shared genes with LSS, the significance proportion of the shared genes is also 0.867. In (C) and (D), the cyan parts represent the point groups' significance proportions, and the red ones represent insignificance proportions. (E) On the base level (the facets Extend), the 87 LSS-Bin-Sig points have much smaller significance p-values than the 18 *groHMM*-Bin-Sig points. If checking the shared genes (Shared-Bin genes) on the base level (the facet Extend), the LSS points also have smaller significance p-values. (F) The LSS-identified points also have much smaller significance p-values than the *MACS2* ones, on the base level (the facet Extend).

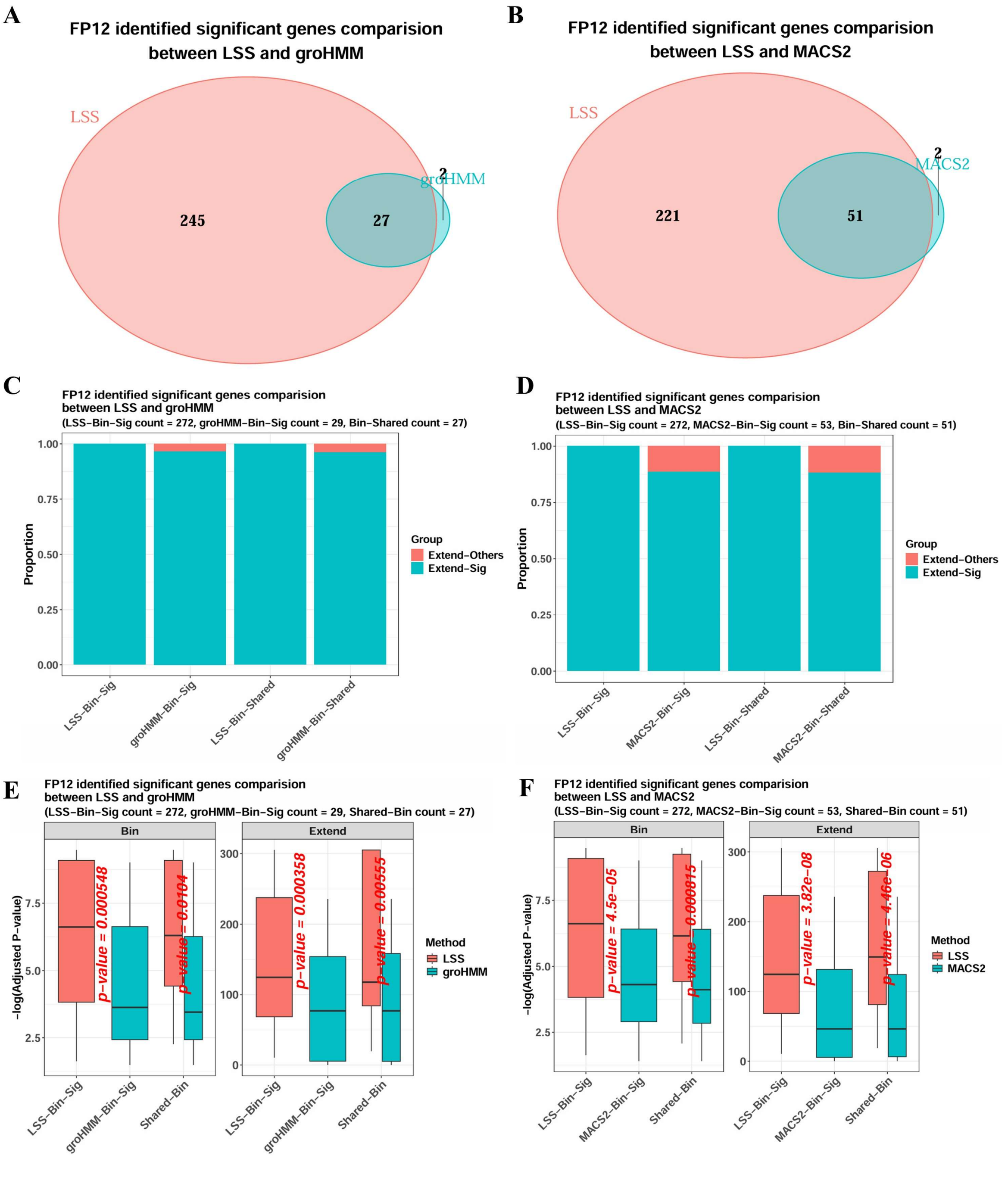

Figure S9. LSS infers the mouse embryonic stem cell transcription rates more accurately than *groHMM* and *MACS2*. (A) In the FP12 data, LSS finds a significant transition point in 272 genes with a length >= 40 kb and an FPKM > 1 in the FP0 reference. However, *groHMM* only identifies a significant point in 29 genes. Among them, 27 genes are shared. (B) On the other hand, *MACS2* identifies a significant point in 53 genes with 51 genes shared. (C) All the

LSS and *groHMM* identified transition points are significant on the bin level. However, if checking them on the base level, all the LSS points, but not all the *groHMM* points, are significant on the base level, and their significance proportions are 1 and 0.966. Moreover, for the shared genes, their points identified by LSS (the LSS-Bin-Shared point group) and the ones identified by *groHMM* (the *groHMM*-Bin-Shared point group) have significance proportions of 1 and 0.963. (D) All the *MACS2* identified transition points are significant on the bin level. However, on the base level, their significance proportion is 0.887. Moreover, for the shared genes with LSS, their points identified by *MACS2* (the *MACS2*-Bin-Shared point group) have a significance proportion of 0.882. In (C) and (D), the cyan parts represent the point groups' significance proportions, and the red ones represent insignificance proportions. (E) On the base level (the facet Extend), the 272 LSS-Bin-Sig points have smaller significance p-values than the 29 *groHMM*-Bin-Sig points. If checking the shared genes (Shared-Bin genes) on the base level (the facet Extend), the LSS points also have smaller significance p-values. (F) On the base level (the facet Extend), the LSS-Bin-Sig points have smaller significance p-values than the *MACS2*-Bin-Sig points. For the shared genes (Shared-Bin genes), on the base level (the facet Extend), the LSS points' significance p-values are also smaller than *MACS2*.

Supplementary Methods

Data collection and preprocessing

The Paf1C, RecQL5, and mouse embryonic stem cell (mESC) data were from GEO (Gene Expression Omnibus) datasets GSE116169 (1), GSE49133 (2), and GSE48895 (3), respectively. Their fastq files were extracted from the corresponding SRA (Short Reads Archive) portal. After quality examination and adaptor trimming with *FastQC* and *Trimmomatic*, they were aligned to mm10 or hg38 reference genomes using *STAR*, and mapped reads were filtered using *SAMtools* so that only the ones with a mapping quality score >= 10 were kept for the downstream analysis.

Transcription rates inference

The function *calrate* in our package was used to infer gene transcription rates. It read in a pair of Pro-seq or Gro-seq bam files: the drug treatment and reference files. The former was from inhibiting transcription for a specific time, and the latter was untreated. Then, for each gene, *calrate* discarded its first and last 1 kb segments, so the remaining part was used to represent the gene, with each base pair in it getting two FPM values from the treatment and reference, respectively. Furthermore, because a gene always had different FPM sum values in the two files, a specific weight was generated for each file and multiply the FPMs there, so that the sum values would be averaged across the files and became the same. In addition, the weights were also used as the gene's pseudo-counts in the files, respectively, to add to their FPMs.

After this normalization, *calrate* divided the gene into 40 bins, and for each bin, its FPM sums in the treatment and reference were calculated, and the ratio between them was obtained. Hence, the gene was converted to a vector with 40 ratios. Then, a pointer scanned through it, and every

time it reached a bin ratio, it divided the whole vector into two segments. Furthermore, for each segment, the variance of its bin ratios was calculated, so a pointer potisoin finally corresponded to two segment variance values, and a sum of squares (SS) value could be obtained by summing them up. Because the pointer scanned through all the 40 bins, a total of 40 SS values would be generated for the gene and the smallest one indicated the treatment file's read-depleted/intact transition point.

Next, the Wilcoxon test was used to check this point's statistical significance. However, at this stage, the point was a bin, including hundreds of bases. Therefore, this bin and its downstream neighbor were merged and expanded to the single-base level, and the above method was used on this expanded region with a pointer going through it base by base. This time, the smallest SS corresponded to the single-base transition point.

The above LSS (least sum of squares) algorithm could be called by setting *calrate*'s parameter *method* as "LSS". On the other hand, if it were set as "HMM", *calrate* would use the HMM (hidden Markov model) algorithm instead but still follow the bin-expansion two-stage framework. After the final transition point was defined, the gene transcription rate could be calculated accordingly.

In addition, the number of bins to be divided for a gene could be set by the parameter *window_num*. Its default value was 40, but the users could change to other bin numbers. Finally, the reason for discarding a gene's first and last 1 kb segments before the inference was to avoid the unstable reads at the transcription starting and ending stages. However, these segments' lengths could be changed via the parameters *startshorten* and *endshorten*. Users could explore more functions of *calrate* via its help document.

Transcription rate inference with *groHMM* and *MACS2*

To use the R package *groHMM* for transcription rate inference (4), we started with the function *windowAnalysis* of this package, which split each bam file into two R vectors. Each of them represented the read counts in a moving window along the plus or minus strand. Hence, for a reference and DRB treatment bam file pair, its two bam files were converted into four R vectors, with each bam generating a plus and a minus strand vector.

Then, we calculated the read ratios for the plus and minus strands, respectively. That is, the treatment and reference vectors of the same strand were used together to calculate a read ratio vector for that strand. Hence, the four vectors above were converted to two, with one corresponding to the treatment/reference read ratios for the plus strand and the other for the minus. During this process, the original read count vectors were normalized with the corresponding bam files' read depths before calculating the ratios.

Finally, the plus and minus strand ratio vectors were transferred to the function *detectTranscripts* in the *groHMM* package to perform the HMM analysis, with default parameters used. This function inferred the transcribed state regions in the genome, and after mapping them to the genes manually, the start point of the most upstream transcribed region within a gene was considered its Pol II-depleted-occupied states transition point, which indicated the transcription distance. After this point was defined, the gene transcription rate was calculated accordingly.

To perform transcription rate inference with *MACS2* (5), the reference and DRB treatment bam files were transferred to the *MACS2 callpeak* function as its peak-calling reference and treatment files, respectively, with other parameters set to their default values. Then, the returned

peak regions were mapped to genes manually and the transition points were identified similarly to the *groHMM* case above.

Simulated data generation

The Paf1C case study's simulated data were generated from the WT0 bam file. Its reads were first filtered to only keep the ones located in 150 pre-selected genes with a length >= 40 kb and an FPKM > 1 in WT0. In addition, to determine these genes, a series of preliminary tests were conducted: all the data from various experimental conditions in the Paf1C dataset GSE116169 were downloaded and transferred to *calrate* to identify transition points with LSS and HMM, and only the genes showing a significant point in >= 2/3 of these tests would be selected, which were the 150 genes. Then, their reads in WT0 were extracted and used to derive various simulations.

At first, a gene's sequencing depth was changed to a pre-defined level. If the new one were less than the original, down-sampling would be used to randomly discard reads to reach the new depth. However, if the new one were greater than the original, up-sampling would be performed via the SMOTE (Synthetic Minority Over-sampling Technique) method. It randomly selected one read in the gene and then searched for its five nearest neighbors. After that, one of them was randomly selected, and its signed distance to the original read was calculated. Then, a number between 0 and 1 was generated from a uniform distribution, and its product with the signed distance was used to shift the neighbor's coordinates, which produced a synthesized read to be added to the gene. This process was repeated until the final sequencing depth reached the expected level.

After that, the gene was divided into two parts according to a pre-defined transition point. The

first part was the read-depleted region, and the other was the intact one. However, if some reads were located across the point, which blurred the regions' boundary, the point and the reads were then attributed to the depleted region, and their downstream part was the intact one. Hence, the final transition point in this case was not the original one but the boundary between the new regions.

Finally, down-sampling was performed within the depleted region according to a pre-defined FPKM ratio between it and the intact one. This ratio ranged from 0.02 to 0.75, so the sampled depleted region always had less FPKM. This stimulated the experimental condition that the DRB treatment inhibited RNA polymerase II (Pol II), making the depleted region's FPKM less than the intact one.

Hence, a gene from the WT0 file was converted following the pre-defined sequencing depth, transition point, and FPKM ratios, and different simulated data were generated accordingly. After that, *calrate* was used to infer the transcription rates from them.

For the RecQL5 and mESC case studies, their simulated data were produced similarly.

To construct the simulated negative datasets with no gene containing a transition point or read-depleted region, the same selected genes in the reference files above were used, and the SMOTE method was performed similarly on them. Then, the synthesized reads from SMOTE and the original reads were used together to perform random sampling to get the simulated data for the same genes. This time, the final simulated data's read depths should be equal to the original data. In addition, no transition point should be defined in the genes, so that no read-depleted regions would be generated by read-down-sampling.

Pause index calculation

The function *calpauseidx* was used to calculate a gene's pause index, which was the ratio of the Pol II density between its promoter and gene body (6). The promoter was defined as the region with a radius of 1 kb around the gene's TSS (transcription start site), and this radius could also be changed by setting the parameter *tssradius* of *calpauseidx*. Correspondingly, the gene body was defined as the region downstream of the promoter. However, if a gene overlapped with others in these regions, it would be removed by *calpauseidx*. Then, the kept genes' reads were extracted from a bam file, which was transferred via the parameter *bamfile*, so that the regions' FPKMs could be obtained and the genes' pause indices could be calculated.

Metagene and gene structure analyses

The function *metaplot* could perform metagene analysis from a single bam file, which was transferred via the parameter *metafile*. It generated the metagene plots for the regions around TSS, around TTS (transcription termination site), and from the upstream of TSS to the downstream of TTS. The parameter *tssradius* defined the TSS region's radius, and *ttsradius* defined that of the TTS region. In addition, *metaplot* compressed all the individual gene regions from TSS to TTS to a unified length so that they could be aligned when plotting the metagene, and the length was set by the parameter *genebodylen*.

In contrast to *metaplot*, which performed on a single bam file, another function, *mmetaplot*, performed on multiple bam files simultaneously. Both of them plotted the metagenes and returned the FPM value on each site. After that, the function *plotprocessing* could accept and further modify these results, such as capturing special metagene parts from them and removing the extremely large FPM values via the parameter *cutoff*, whose default was 0.01, meaning the 99% quantile of the metagene FPMs would be defined as their maximum, and any larger ones

would be reduced.

For the gene structure analyses, such as calculating the gene GC contents and exon densities, and performing *k*-mer analysis, they were conducted by the package's functions *getgc*, *getexon*, and *getkmer*. In addition, the GC contents and exon densities could also be analyzed by *calrate* during its rates inference and returned with the rates results.

Gene functional enrichment analysis

For the faster and slower genes identified in the FP case study, their enriched functions were checked via the package *enrichR* on the databases "BioPlanet_2019" and "Reactome_2016" (7).

References

1. Hou, L., Wang, Y., Liu, Y., Zhang, N., Shamovsky, I., Nudler, E., Tian, B. and Dynlacht, B.D. (2019) Paf1C regulates RNA polymerase II progression by modulating elongation rate. *Proceedings of the National Academy of Sciences*, **116**, 14583-14592.
2. Saponaro, M., Kantidakis, T., Mitter, R., Kelly, Gavin P., Heron, M., Williams, H., Söding, J., Stewart, A. and Svejstrup, Jesper Q. (2014) RECQL5 Controls Transcript Elongation and Suppresses Genome Instability Associated with Transcription Stress. *Cell*, **157**, 1037-1049.
3. Jonkers, I., Kwak, H. and Lis, J.T. (2014) Genome-wide dynamics of Pol II elongation and its interplay with promoter proximal pausing, chromatin, and exons. *eLife*, **3**, e02407.
4. Chae, M., Danko, C.G. and Kraus, W.L. (2015) groHMM: a computational tool for identifying unannotated and cell type-specific transcription units from global run-on sequencing data. *BMC Bioinformatics*, **16**, 222.
5. Feng, J., Liu, T., Qin, B., Zhang, Y. and Liu, X.S. (2012) Identifying ChIP-seq enrichment using MACS. *Nature Protocols*, **7**, 1728-1740.
6. Williams, Lucy H., Fromm, G., Gokey, Nolan G., Henriques, T., Muse, Ginger W., Burkholder, A., Fargo, David C., Hu, G. and Adelman, K. (2015) Pausing of RNA Polymerase II Regulates Mammalian Developmental Potential through Control of Signaling Networks. *Molecular Cell*, **58**, 311-322.
7. Kuleshov, M.V., Jones, M.R., Rouillard, A.D., Fernandez, N.F., Duan, Q., Wang, Z., Koplev, S., Jenkins, S.L., Jagodnik, K.M., Lachmann, A. *et al.* (2016) Enrichr: a comprehensive gene set enrichment analysis web server 2016 update. *Nucleic acids research*, **44**, W90-97.
